# A landscape of X-inactivation during human T cell development

**DOI:** 10.1038/s41467-024-54110-7

**Published:** 2024-12-04

**Authors:** Björn Gylemo, Maike Bensberg, Viktoria Hennings, Christina Lundqvist, Alessandro Camponeschi, Dóra Goldmann, Huan Zhang, Aida Selimović-Pašić, Antonio Lentini, Olov Ekwall, Colm E. Nestor

**Affiliations:** 1 Crown Princess Victoria Children’s Hospital, and Department of Biomedical and Clinical Sciences (BKV), Linköping University, Linköping, Sweden; 2 Department of Rheumatology and Inflammation Research, Institute of Medicine, The Sahlgrenska Academy, University of Gothenburg, Gothenburg, Sweden; 3 Department of Pediatrics, Institute of Clinical Sciences, The Sahlgrenska Academy, University of Gothenburg, Gothenburg, Sweden

**Keywords:** Autoimmunity, Dosage compensation, T cells, Systems analysis

## Abstract

Females exhibit a more robust immune response to both self-antigens and non-self-antigens than males, resulting in a higher prevalence of autoimmune diseases but more effective responses against infection. Increased expression of X-linked immune genes in female T cells is thought to underlie this enhanced response. Here we isolate thymocytes from pediatric thymi of healthy males (46, XY), females (46, XX), a female with completely skewed X-chromosome inactivation (46, XX, cXCI) and a female with Turner syndrome (45, X0). Using whole exome sequencing, RNA sequencing and DNA methylation data, we present a sex-aware expression profile of T cell development and generate a high-resolution map of escape from X-chromosome inactivation (XCI). Unexpectedly, XCI is transcriptionally and epigenetically stable throughout T cell development, and is independent of expression of *XIST*, the lncRNA responsible for XCI initiation during early embryonic development. In thymocytes, several genes known to escape XCI are expressed from only one X-chromosome. Additionally, we further reveal that a second X-chromosome is dispensable for T cell development. Our study thus provides a high-resolution map of XCI during human development and suggests a re-evaluation of XCI in sex differences in T cell function.

## Abstract (web summary)

X-chromosome inactivation (XCI) contributes to sex bias in T cell immunity, but data on profiling XCI during human T cell development is still lacking. Here, the authors leverage allele-specific expression, sex-biased gene expression and DNA methylation data on human pediatric thymocytes to find surprisingly stable XCI during thymocyte differentiation.

## Introduction

T cells are a crucial component of adaptive immunity in humans. Although the anatomy of T cell development is identical in males and females, females tend to have a more robust immune response to antigens and higher resistance to both viral and bacterial infections than males^1,2^. The latter has been emphasized by the COVID-19 pandemic, which has resulted in greater mortality in men^3^. While it is possible that differing exposure and infection rates have impacted this observation, it has also been associated with sex-specific differences in the immune response to SARS-CoV-2 infection, specifically in T cell responses^3^. Indeed, females have higher levels of mature CD4 + T cells and generate more activated T cells following in vitro stimulation^2–6^. In addition, females are more susceptible to autoimmune diseases, with many, including multiple sclerosis (MS) and systemic lupus erythematosus (SLE), exhibiting a profound female bias (>3 female:1 male)^7^ suggestive of a more hyper-responsive adaptive immune system in females compared to males. This sexual dimorphism of immune system function has been linked to (i) hormonal differences, (ii) divergence of thymic T cell development and selection, and (iii) the presence of an additional, albeit inactivated, X-chromosome in all female cells^2,8^.

Whereas female (XX) cells undergo X-inactivation (XCI) during embryonic development to balance chromosomal dosage against males (XY)^9^, around 15–20% of X-linked genes in humans escape silencing and remain biallelically expressed^10,11^ resulting in higher levels of gene products in female cells. Importantly, several of these proposed escapee genes have a known role in the adaptive immune response (e.g., *CD40LG*, *TLR7* and *CXCR3*), implicating the immune-modulatory potential of X-chromosome dosage as a source of adaptive immune response differences between males and females^12^. Indeed, males with Klinefelter syndrome (KS) carry an additional X-chromosome (47, XXY) and have an increased risk for autoimmune disease compared to males of normal karyotype (46, XY). Similarly, expression of *TLR7* and *CD40LG* in activated CD4+ T cells is higher in females and KS males than in males of normal karyotype (46, XY) or Turner syndrome (TS) females lacking a second X-chromosome (45, X0)^6^. Finally, recent studies have revealed that *XIST* coating of the inactive X-chromosome is dynamic during normal T cell development in mouse and human and suggested that re-activation of silenced X-linked genes may be required for appropriate T cell development^13,14^.

Although recent studies have advanced our understanding of the trajectory of human thymocyte development^15,16^, they have not addressed the sex-specific characteristics of T cell development, which remains largely unexplored.

Here, using thymocyte subpopulations isolated from healthy male and female pediatric thymi, we specifically aim to reveal the sex-specific features of human thymocyte development and whether they could underlie the difference in T cell biology observed between males and females. Using pediatric female samples 100% skewed for X-inactivation (cXCI) or from a TS patient we map the XCI landscape and functionally dissect the contribution of the inactive X-chromosome to T cell development in humans. We find escape from XCI to be highly stable across thymocyte populations and reveal that the second X-chromosome in females does not contribute to proper T cell development. This work motivates a re-assessment of XCI during pediatric T cell development as the origin of sex differences in T cell function.

## Results

### A unique landscape of sex-biased gene expression in human thymocytes

**Fig. 1:**
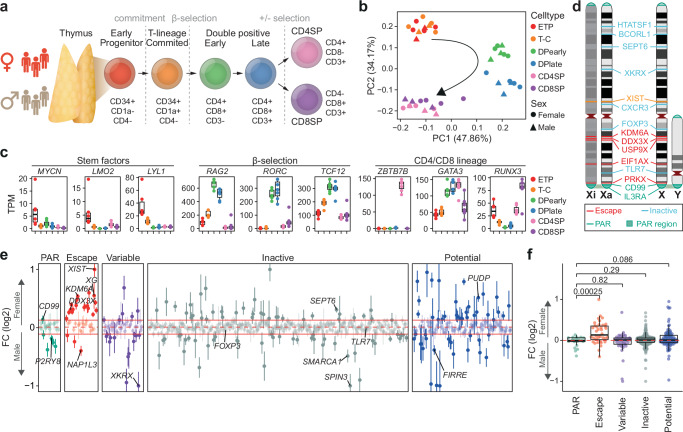
Sex-biased escape gene expression in human thymocytes. **a** Thymocyte development and markers used for cell sorting. **b** Principal component analysis (PCA) of normalized RNA-seq counts from male (triangle) and female (circle) developing thymocytes. **c** Expression dynamics as transcript per million (TPM) of genes associated with key transitions during thymocyte development. Boxplot representing median (central line), first and third quartiles (Q1 and Q3, respectively) (box edges) and 1.5*inter quartile range (IQR) from Q1 and Q3 (whiskers) from seven biological replicates (3 males and 4 females) are shown. **d** Schematic representation of selected genes escaping and inactivated by X-chromosome inactivation on the active (Xa) and inactive (Xi) X-chromosome. **e** Sex-biased expression of genes across the X-chromosome as log2 fold change (FC) of expression in female over male thymocytes. Horizontal red lines indicate median (dotted line) ± IQR of inactive genes (filled lines). FC (log2) values have been capped to 1 or −1 if above or below ±1, respectively. FC (log2) (dots) and standard error (vertical lines) of seven thymocyte populations from females (*n* = 4) and males (*n* = 3) shown. **f** Sex-biased gene expression across the X-chromosome as log2 FC of expression in female over male thymocytes by known XCI status. *P*-values from Benjamini-Hochberg (BH) corrected two-tailed T-test. Boxplot representing median (central line), Q1 and Q3 (box edges) and 1.5*IQR from Q1 and Q3 (whiskers) across all genes. Dots representing mean of six thymocyte populations from seven biological replicates (3 males and 4 females). **e**, **f** XCI status defined based on previous assessment^10^ with potential XCI escape genes (blue) previously not investigated or classified as unknown. **a**, **b**, **c** ETP, early T cell progenitors; T-C, T cell committed thymocytes; DPearly, early double positive thymocytes; DPlate, late double positive thymocytes; CD4SP, CD4 single positive thymocytes; CD8SP, CD8 single positive thymocytes. **d**, **e**, **f** PAR, pseudoautosomal region. Source data are provided as a Source Data file.

T cell development in the thymus occurs in a tightly controlled stepwise manner^15,17^. To establish a sex-resolved transcriptomic map spanning thymic T cell development, we performed mRNA sequencing (RNA-seq) on thymocyte populations corresponding to key developmental stages from healthy male (*N* = 3) and female (*N* = 4) thymi (Fig. 1a, Supplementary Fig. 1, Supplementary Table 1 & Supplementary Table 2). The six selected thymocyte subsets are well characterized and capture the key steps in human T cell development^16,18^. Principal component analysis of global expression revealed distinct profiles for each subset matching their developmental trajectories (Fig. 1b & Supplementary Fig. 2a, b). In line with previous studies^15,18^, modeling of differential expression across cell types revealed enrichment for genes involved in T cell selection and cell-type specific expression of key genes involved in early thymic progenitor (ETP) stemness, CD4 and CD8 double positive (DP) cell β-selection and CD4 or CD8 single positive (SP) cell lineage commitment^19^ (Fig. 1c, Supplementary Data 1, Supplementary Data 2). These results highlight not only the coordinated temporal regulation of human thymocyte development but also the specific checkpoint-associated changes (Supplementary Fig. 2c, d**)**.

In line with expression of 100-150 escape genes from the inactive X-chromosome (Xi) in females (Fig. 1d), chromosome X had the highest proportion of genes showing significant sex-biased expression (Wald test, *P* < 1 × 10^−5^ & absolute log2FC > 0.2) during thymocyte development (Fisher’s Exact Test, *P* = 1.744 × 10^−6^) (Supplementary Fig. 3a, Supplementary Data 3 & Supplementary Data 4). Many well-characterized escape genes (e.g., *CDK16, DDX3X, EIF1AX, EIF2S3, FUNDC1, JPX, KDM5C, KDM6A, PRKX, PUDP, SMC1A, STS, UBA1, ZFX, ZRSR2*) exhibited a pronounced female-biased expression, reflecting biallelic expression in females and subtype expression in males (Fig. 1e, f). Female-biased escape genes were expressed 0.22-0.59 times higher in females than males (Wald test, *P* < 1 × 10^−5^), consistent with the observation that expression of transcripts from the Xi is typically lower than their expression from the active X-chromosome (Xa) (Fig. 1f). Interestingly, we were unable to confirm female sex-biased expression in a number of known escape genes *(AP1S2*, *ARSD*, *CXorf38*, *GEMIN8*, *GPM6B*, *IKBKG*, *MED14*, *MSL3, NAP1L3*, *OFD1*, *RAB9A*, *RENBP*, *SYAP1*, *TCEANC*, *TRAPPC2*, *TXLNG*, *USP9X;* Wald test, *P* > 1 × 10^−5^ & log2FC < 0.2) (Fig. 1e). One gene assumed to undergo XCI showed a significant (Wald test, *P* < 1 × 10^−5^ & log2FC < −0.2) male-bias (*SPIN3*).

**Fig. 2:**
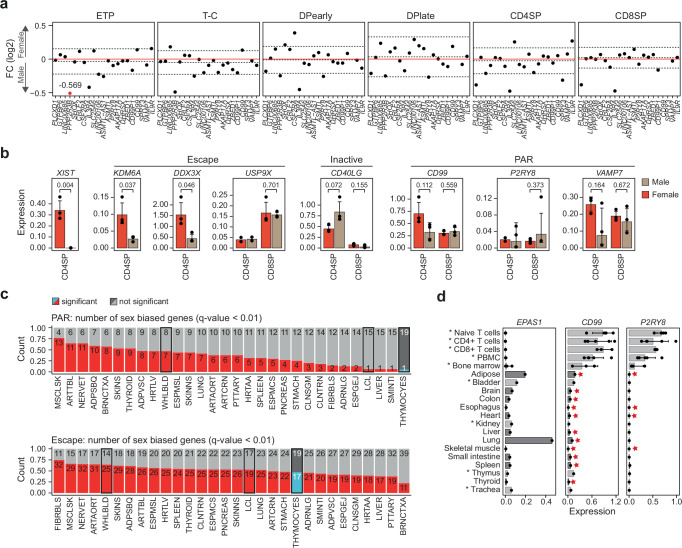
Unique expression of PAR genes in human thymocytes. **a** Sex-biased expression of genes in the pseudoautosomal region (PAR) as log2 fold change (FC) in the six thymocyte subpopulations. Dashed lines indicate median ± IQR for each cell type. Red dots represent FC (log2) values outside the −0.5:0.5 range that were capped to −0.5 or 0.5 FC (log2) with exact values indicated for each capped data point. ETP, early T cell progenitors; T-C, T cell committed thymocytes; DPearly, early double positive thymocytes; DPlate, late double positive thymocytes; CD4SP, CD4 single positive thymocytes; CD8SP, CD8 single positive thymocytes. **b** Expression in male (brown) and female (red) CD4 and CD8 single positive (SP) thymocytes of a selection of escape, inactive and PAR genes analyzed by qPCR normalized to *GAPDH*. Bars indicate mean expression of biological replicates and hinges standard deviation. *P*-values comparing expression in males and females indicated above; unpaired, two-tailed student t-test. Standard deviation and *p*-value only shown where 3 biological replicates were available in each group. **c** Number of significant sex-biased PAR (upper) and escape genes (lower) in 30 different tissues, comparing thymocytes to GTEx data processed in Tukiainen et al.^10^. Statistical significance for thymocytes: Wald test (BH corrected) *P*-value < 0.01, for GTEx data eBayes FDR < 0.01. Hematopoietic samples (whole blood, EBV-transformed lymphocytes and thymocytes) are highlighted. Tissue abbreviations can be found in Supplementary Table 3. **d** Gene expression of *EPAS1*, *CD99* and *P2RY8* across an independent set of 20 tissues analyzed by qPCR and normalized to *GAPDH* expression. Bars indicate mean expression and hinges standard deviation. Stars highlight tissues that have previously been shown to be sex-biased^10^. Asterix and different nuances of gray depict tissues that were included (dark gray) or were not included (light gray, asterisk) in the analysis of sex-biased expression in GTEx shown in panel c. PBMC, peripheral blood mononuclear cells. Source data are provided as a Source Data file.

Approximately 3% of X-linked genes are located in the pseudoautosomal region (PAR) at the tips of the q-arm (~200 Mbp) and p-arm (~350 kbp) of chromosome X, which are homologous to PARs on the Y-chromosome (Fig. 1d). As expression from the Xi is typically lower than that observed from the Xa^10,20^, combined expression of alleles in the PAR region in females is assumed to be lower than that of the same alleles from the X and Y PAR regions in males, resulting in male-biased expression of PAR genes. However, we observed no consistent male-bias in PAR gene expression in thymocytes (Figs. 1f, 2a), which was confirmed by qPCR in CD4SP and CD8SP cells (Fig. 2b). Interestingly, the gene *PPP2R3B* showed a distinct subtype specific pattern of male-biased expression in ETP and T cell committed (T-C) cells (Fig. 2a). Protein phosphatase 2 (PP2A), of which PPP2R3B is a subunit, is a powerful negative regulator of cell proliferation, has been shown to play a key role in mammalian thymocyte development and is required for appropriate thymocyte selection and DP cell maturation^21–23^. Nonetheless, the general lack of male-biased PAR gene expression was unexpected and in stark contrast to a previous study of X-linked gene expression, which reported consistently higher expression of PAR genes in males across 29 tissues in the GTEx database^10^. Upon meta-analysis of the GTEx data we observed the expected, consistent female-biased expression of nonPAR escape genes but found sex-bias of PAR genes to be infrequent and highly tissue specific (Fig. 2c). Interestingly, the sole lymphocyte sample (LCL) showed the lowest number of sex-biased PAR genes (*N* = 1), similar to thymocyte samples analyzed here (Fig. 2c). Analysis of expression of two PAR genes previously reported to show pronounced male biased expression (*CD99*, *P2RY8*)^10^ by qPCR in an independent panel of 20 human tissue pools revealed low or absent expression of these genes in many solid tissues (Fig. 2d) suggesting that detection of male sex-bias in some tissues was artefactual, resulting from low levels of expression. Indeed, expression of *CD99* and *P2RY8* as detected by qPCR in the majority of tissues was below expression of *EPAS1*, a gene expressed specifically in adipose, lung and placental tissue (Fig. 2d). Failure to account for the duplication of X and Y PAR regions in reference genomes during alignment can also result in inaccurate estimation of gene expression^24^. Comparing the sex-biased gene expression obtained with a pseudo-aligner (Salmon) to that obtained from a reference-based alignment method (STAR), the pseudo-aligner showed a weaker shift towards male and female bias (Supplementary Fig. 3b, Supplementary Data 5 & Supplementary Data 6). Indeed, re-evaluation of X-linked expression using pseudo-alignment in 46 tissues in 898 individuals showed reduced sex-bias in both PAR (male bias) and escape genes (female bias) (Supplementary Fig. 3c, d & Supplementary Data 7). Importantly, sex-biased expression of PAR genes was highly tissue specific with adipose tissue and thymocytes showing no bias, whereas pronounced male-biased expression was observed in brain (Supplementary Fig. 3c). To further exclude alignment-related artefacts, we re-analyzed expression microarray data available from GTEx, which again confirmed low levels of sex-biased expression of PAR genes across most tissues (Supplementary Fig. 3e & Supplementary Table 3).

Together, our findings reveal a distinctly unique pattern of sex-biased gene expression in developing human T cells and that previous estimates of sex-biased expression of X-linked genes may have markedly overestimated this phenomenon.

### Stable X-inactivation during human T cell development

**Fig. 3:**
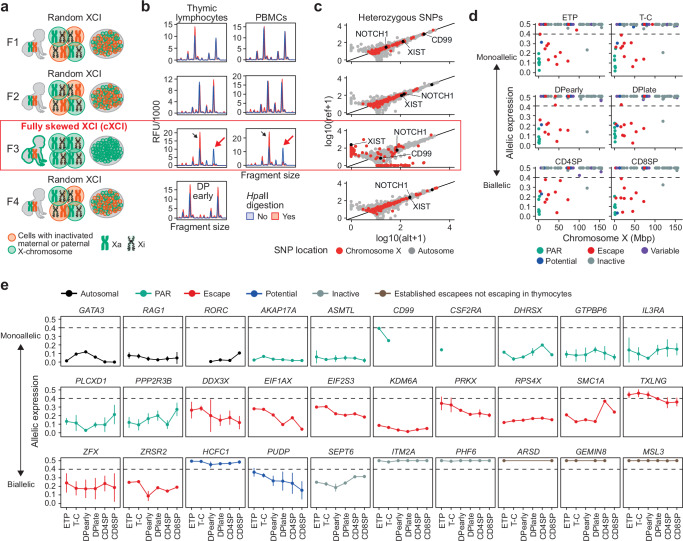
X-inactivation dynamics during human T cell development. **a** Overview of X-chromosome inactivation (XCI) skewing in females included in the thymocyte data set with schematic representation of active (Xa) and inactive (Xi) X-chromosomes. **b** HUMARA assay of Female 1 (F1), F2 and F3 in thymic lymphocytes and peripheral blood mononuclear cells (PBMCs) and F4 in early double positive (DPearly) cells. Black arrows show peak detection in digested and undigested DNA and red arrows show complete loss of one allele after *HpaII* digestion (red) indicating complete XCI skewing (cXCI). **c** Allele-specific expression (ASE) of genes with heterozygous SNPs (hSNP) as expression from the reference (ref) and alternative (alt) allele from chromosome X and autosomal genes in F1, F2, F3 and F4. **d** ASE, chromosomal location and escape status of expressed X-chromosome genes with at least one hSNP in F3. **e** ASE of a selection of genes across thymocyte development in F3 (*n* = 1 for each thymocyte subpopulation). Dots and lines indicate mean and standard deviation, respectively. **d**, **e** XCI status defined based on previous assesment^10^ with potential XCI escape genes (blue) previously not investigated or classified as unknown. Source data are provided as a Source Data file.

During quality control of the thymocyte RNA-seq data we observed that the majority of reads mapping to the X-chromosome in a single female sample (F3) were transcribed from the same copy of the X-chromosome, as evidenced by a lack of heterozygous single nucleotide polymorphisms (SNPs) in randomly inactivated X-linked genes (Supplementary Fig. 4a). Detection of subtype expression in bulk tissue samples is only possible in karyotypically normal females exhibiting complete skewing of X-inactivation (cXCI); the same parental X-chromosome has been inactivated in every cell in the examined tissue (Fig. 3a). Complete XCI skewing in sample F3 was confirmed by HUMARA assay (Fig. 3b)^25^ and further verified by whole exome sequencing (WES) followed by allele-specific expression analysis of all four female samples (Fig. 3c & Supplementary Fig. 4b, c). This powerful natural genetic model allowed allele-specific expression mapping throughout human thymocyte development including 127 X-linked genes for which we detected 259 heterozygous SNPs in F3 (filters: WES, read count >20 for both alleles, RNA-seq readcount >10, and minor allele frequency (MAF) > 10% in either RNA-seq or WES) (Fig. 3d & Supplementary Fig. 4b, c). In F3, PAR genes were expressed from both X-chromosomes whereas genes undergoing XCI showed consistent monoallelic expression (allelic expression >0.4) (Fig. 3d, e & Supplementary Fig. 5). Our unique data allowed us to build on our landscape of sex-biased expression and confirm escape status for novel (*PUDP*, *SEPT6*) and known (*DDX3X*, *EIF1AX*, *EIF2S3*, *KDM6A*, *PRKX*, *RPS4X*, *SMC1A*, *TXLNG*, *ZFX*, *ZRSR2*) escape genes in thymocytes (Fig. 3e & Supplementary Data 7).

**Fig. 4:**
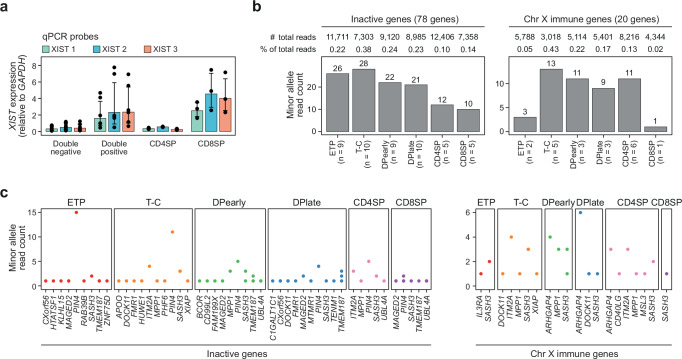
Stable expression from the inactive X-chromosome. **a** *XIST* expression in double negative (ETP, T-C), double positive (DPearly, DPlate), CD4SP and CD8SP cells based on qPCR for 3 separate qPCR probes (Supplementary Table 5). *XIST* expression normalized to expression of *GAPDH*. Bars indicate mean expression and hinges standard deviation from six biological replicates for each of the six thymocyte subpopulations. **b** Total minor allele read count for heterozygous SNPs in all inactive (left) and immune genes (right) in F3 (1 biological replicate for each thymocyte subpopulation). *n*, number of inactive or immune genes expressed from Xi in each cell type. **c** Minor allele read count of individual inactive or immune genes on chromosome X that are expressed from the inactive X-chromosome (Xi) in F3 (*n* = 1 for each thymocyte subpopulation). See “methods” for immune gene definitions. **a**, **b**, **c** ETP, early T cell progenitors; T-C, T cell committed thymocytes; DPearly, early double positive thymocytes; DPlate, late double positive thymocytes; CD4SP, CD4 single positive thymocytes; CD8SP, CD8 single positive thymocytes. Source data are provided as a Source Data file.

Our finding of stable subtype expression of inactivated genes throughout T cell development (Fig. 3d, e & Supplementary Fig. 5) is at odds with the emerging paradigm of low fidelity (leaky) XCI maintenance during lymphocyte development in females resulting in atypical expression across the X-chromosome and ultimately altered T cell function between the sexes^13,14,26,27^. Notably, the stability of Xi gene repression during thymocyte development contrasted with highly variable expression of *XIST* between different T cell sub-populations including a 7-fold expression difference between CD4 and CD8 single positive T cells (Fig. 4a). Despite the profound variability in *XIST* expression, no significant variability in minor (Xi) allele expression at inactive genes^10^ (*N*_*GENES*_ = 78; *N*_*HET-SNPS*_ = 145) was observed (Fig. 4b, c), decoupling *XIST* expression levels from XCI maintenance during thymocyte development. A similar lack of minor allele expression was observed across X-linked immune genes^28–30^ (*N*_*GENES*_ = 20; *N*_*HET-SNPS*_ = 40); < 0.5% of total reads (Fig. 4b, c). Importantly, the vanishingly low levels of minor allele expression are inconsistent with complete loss of XCI in even a small sub-population (2–5%) of the total T cell pool, as previously proposed^13,14^.

**Fig. 5:**
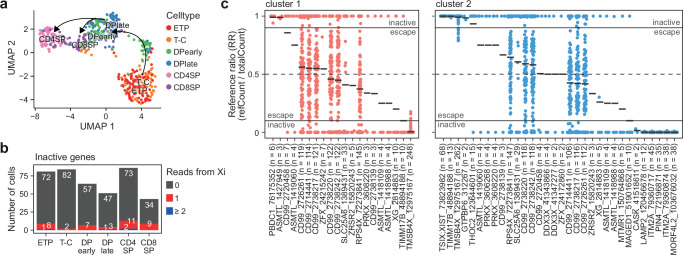
Single cell sequencing of human thymocyte populations. **a** UMAP visualization of thymocyte subpopulations from Smart-seq2 data. **b** Number of cells with 0, 1 or ≥ 2 reads from inactive genes on the inactive X-chromosome (Xi). **c** Reference ratio (RR, reference read count / total read count) of heterozygous SNP expression from single cells pooled based on clusters identified in Supplementary Fig. 6. Black lines indicate mean RR per gene and cluster. Only SNPs with RR not equal to 0 or 1 shown. Escape from X-inactivation: reference ratio: 0.1–0.9. *n*, number of cells for which expression of the SNP was detected. Numbers after gene symbols are the genomic position of the heterozygous SNP. Source data are provided as a Source Data file.

To further validate our findings, we performed single cell whole transcript sequencing (Smart-seq2) of sorted thymocytes from a non-skewed female thymus (F4, Fig. 3a–c). Approximately 0.4 million aligned reads were obtained per cell for 69-84 cells per subtype (Supplementary Table 2). Clustering revealed a highly similar developmental trajectory of thymocyte development to that observed from bulk RNA-seq analyzes (Fig. 5a). Whole exome sequencing identified 206 heterozygous SNPs in 115 X-linked genes allowing more accurate classification of allelic expression (Supplementary Data 8). The number of reads arising from the Xi at inactive genes was low in all subtypes consistent with XCI remaining highly stable throughout human T cell development (Fig. 5b).

We further leveraged the single cell RNA-seq data to investigate the XCI status of individual genes using a pseudo-bulk approach to infer the inactive X-chromosome in each cell^31,32^. Allelic *XIST* expression showed a clear distinction between cells expressing reference or alternative allele (Supplementary Fig. 6a) and clustering *XIST* expressing cells on reference ratio of all X-linked genes resulted in two clear clusters of cells reflecting the expression of SNPs arising from either the paternal or maternal X-chromosome (Supplementary Fig. 6b). As *XIST* read counts were only obtained in 150/516 cells, the allelic expression of seven additional inactive genes allowed us to infer Xa and Xi in the remaining 366 cells (Supplementary Fig. 6c & Supplementary Data 9). Importantly, cells of all 6 thymocyte subtypes followed the same clustering pattern (Supplementary Fig. 6b), once again suggesting that a widespread increase in biallelic expression from the X-chromosome does not occur during T cell development in humans.

Finally, we leveraged information on Xi-clustering to determine XCI status of 132 heterozygous SNPs in 83 X-linked genes (Fig. 5c & Supplementary Fig. 6d). In cluster 1, 17 out of 107 (15.8 %) heterozygous SNPs and 9/72 (~ 12.5 %) genes escaped XCI while in cluster 2 the proportion of escaping heterozygous SNPs were 21/104 (20 %) and escaping genes 11/67 (16.4 %). Out of the 79 heterozygous SNPs that were identified in both clusters, 73 were classified with the same XCI status while six heterozygous SNPs (*DDX3X* (chrX:41,343,866), *MAGED1* (chrX:51,901,652), *PRKX* (chrX:3,606,268), *TIMM17B* (chrX:48,894,188), *ZFX* (chrX:24,172,860) and *ZFX* (chrX:24,215,242)) were classified differently between clusters (Fig. 5c & Supplementary Fig. 6d).

Taken together, our results exclude widespread atypical biallelic expression caused by a general relaxation of XCI during human thymocyte development. However, our results do not exclude locus-specific loss of XCI resulting in biallelic expression of specific genes as reported for some X-linked immune genes such as *TLR7*^33^.

### A refined landscape of X-inactivation in humans

**Fig. 6:**
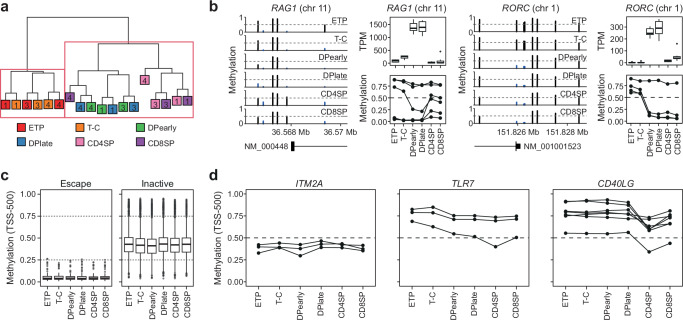
DNA methylation on chromosome X is stable during T cell development. **a** Hierarchical clustering of the 1000 most variable methylated sites in EPIC array methylation data from thymocyte populations in females F1, F3, and F4. Individuals indicated as numbers. **b** Methylation around transcription start sites (TSS) (left), gene expression as transcript per million (TPM) (top) and methylation of probes −500 and +1500 from TSS (bottom) in thymocyte subpopulation for genes involved in thymocyte development (*RAG1* and *RORC*). Boxplot representing median (central line), first and third quartiles (Q1 and Q3, respectively) (box edges) and 1.5*inter quartile range (IQR) from Q1 and Q3 (whiskers) from three biological replicates (all female) are shown. **c** Boxplot representing median (central line), first and third quartiles (Q1 and Q3, respectively) (box edges) and 1.5*inter quartile range (IQR) from Q1 and Q3 (whiskers) of DNA methylation of all probes in −500 TSS range of escape (left) or inactive genes (right) from three biological replicates (all female), including only genes that are found to have the same XCI status in thymocytes and previous assessments^10^. Dashed lines highlight low (methylation ≥0.25), intermediate (0.26-0.75) and high ( > 0.76) DNA methylation. **d** DNA methylation at TSS −500 across thymocyte development for *ITM2A*, *TLR7* and *CD40LG*. **a**, **b**, **c**, **d**, ETP, early T cell progenitors; T-C, T cell committed thymocytes; DPearly, early double positive thymocytes; DPlate, late double positive thymocytes; CD4SP, CD4 single positive thymocytes; CD8SP, CD8 single positive thymocytes. Source data are provided as a Source Data file.

The direct determination of allelic expression was limited to genes carrying a heterozygous SNP, representing less than 25% of all expressed X-linked genes. To estimate chromosome-wide levels of XCI we mapped DNA methylation in all six thymocyte subtypes in three female samples (F1, F3 and F4) using EPIC methylation arrays. Analysis of the 1000 most variable CpGs resulted in clustering of samples based on developmental stage (Fig. 6a) in a pattern highly similar to that obtained with RNA-seq (Fig. 1b). As expected, expression changes of many key thymocyte transition genes (e.g., *RAG1*, *RORC*, *CD8A*, *CD3D, CD3G*) were accompanied by corresponding changes in promoter DNA methylation (Fig. 6b & Supplementary Fig. 7a). Consistent with stable allelic expression throughout thymocyte development, irrespective of *XIST* expression, no pronounced global alterations in DNA methylation on the X-chromosome were observed (Supplementary Fig. 7b, c).

Escape genes with confirmed biallelic expression were typified by a lack of promoter methylation throughout development whereas confirmed inactive genes were characterized by intermediate (25–75% methylation) levels of promoter methylation (Fig. 6c). As observed for gene expression levels, DNA methylation at inactive genes was highly stable throughout thymocyte development and showed no association with levels of *XIST* expression (Supplementary Fig. 7d), further de-coupling *XIST* from maintenance of XCI in thymocytes. More specifically, genes previously reported to show leaky expression from the Xi during lymphocyte development (*CD40LG*, *TLR7, ITM2A*) also showed no association between *XIST* expression and promoter methylation (Fig. 6d).

**Fig. 7:**
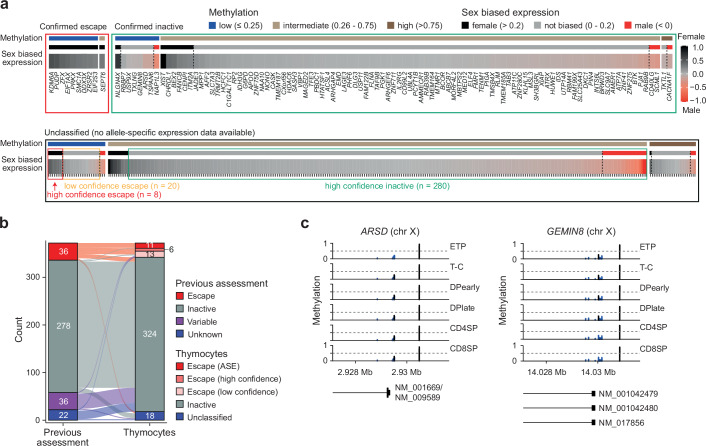
X-inactivation during T cell development. **a** X-inactivation escape status as inactive (green box) or escape (red and orange box), DNA methylation (low, intermediate or high) and sex-biased expression as fold change (FC) (log2) in females over males of all expressed chromosome X genes in thymocytes. Confirmed escape status is inferred from F3 allele-specific expression analysis (inactive genes = mean allele-specific expression across thymocyte development (ASE) >0.4, escape genes = ASE < 0.4). DNA methylation for each gene as mean methylation of −500 TSS probes categorized into low (methylation ≤0.25), intermediate (0.26–0.75) and high ( >0.75) methylation. High confidence escape genes: low promoter methylation and sex-biased expression > 0.2; low confidence escape genes: low promoter methylation and sex biased expression 0-0.2; high confidence inactive genes: intermediate or high methylation and sex-biased expression < 0.2. Genes that fall outside of these thresholds are considered unclassified. See “methods” for details. **b** Escape status comparison between previous escape assessment^10^ and our new classification in thymocytes. Calls as escape genes were either based on allele-specific expression (ASE) or as high/low confidence according to panel a. **c**, DNA methylation of probes around transcription start sites of X-chromosome genes, *ARSD* and *GEMIN8*. ETP, early T cell progenitors; T-C, T cell committed thymocytes; DPearly, early double positive thymocytes; DPlate, late double positive thymocytes; CD4SP, CD4 single positive thymocytes; CD8SP, CD8 single positive thymocytes. Expression data from seven biological replicates (4 female, 3 male) and DNA methylation data from three female biological replicates. Source data are provided as a Source Data file.

Leveraging our allelic expression data, we defined the sex-biased expression and DNA methylation characteristics of canonical escape and inactive genes (Fig. 7a, upper panel). Using this classification, we identified a further 21 and 291 putative escape and inactive genes, respectively (Fig. 7a, lower panel & Supplementary Data 10). 86% (288/334) of the predicted genes agreed with XCI gene classifications from previous studies, confirming the accuracy of our approach (Fig. 7b & Supplementary Data 11). Moreover, several genes that were re-classified here from escape to inactive (e.g., *GPM6B*, *IKBKG*) have recently been reported as having atypically high levels of promoter DNA methylation^34^, consistent with genes undergoing XCI. In total, we re-classified the XCI status of 62 genes in T cells (Supplementary Data 11).

Importantly, we also identify three genes previously described as escapees that did not escape in thymocytes (*ARSD*, *GEMIN8* & *MSL3*) (Fig. 3e). The promoters of both *GEMIN8* and *ARSD* were unmethylated (Fig. 7c), typical of escape genes, but neither showed female-biased expression (Supplementary Data 10), reinforcing the need to combine both data types for accurate XCI classification. Interestingly, mutations in *MSL3* were recently discovered as the genetic cause of a novel neurodegenerative disease, Basilicata-Akhtar syndrome^35^. Indeed, the lack of sex-biased expression of *MSL3* across multiple tissues and intermediate levels of DNA methylation (Supplementary Fig. 7e) are also consistent with its classification as an inactive gene, with no evidence of female biased expression in brain, the primary affected organ (Supplementary Fig. 7f). Importantly, re-classification of *MSL3* as an inactive gene precludes escape from inactivation as an explanation for the equal penetrance of Basilicata-Akhtar syndrome in affected males and females^35,36^.

In conclusion, by combined allele-specific expression, DNA methylation and sex-biased expression analysis we classify the XCI status of ~73% (354/484) of expressed X-linked non-pseudo genes in thymocytes, representing the most comprehensive map of XCI in any human tissue to date.

### The inactive X-chromosome does not contribute to T cell development

**Fig. 8:**
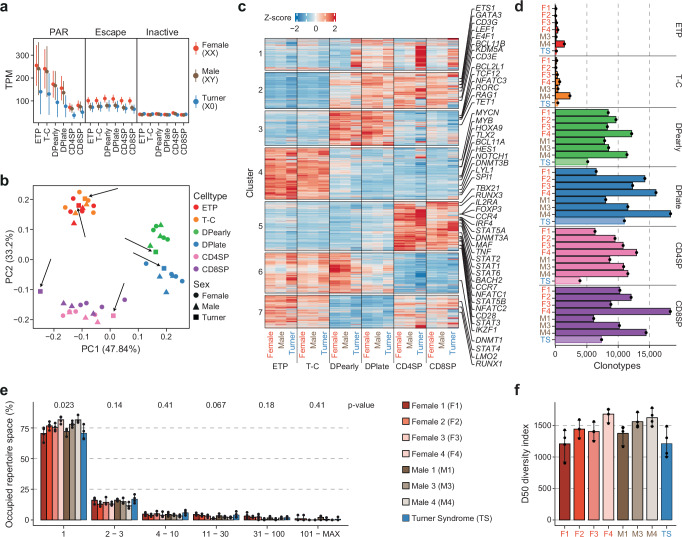
The inactive X-chromosome does not contribute to T cell development in humans. **a** Expression as transcript per million (TPM) of genes in PAR (pseudoautosomal region), escape and inactive genes for each thymocytes subpopulation in XX females (orange, 4 biological replicates), XY males (brown, 3 biological replicates) and X0 Turner syndrome patient (blue, 1 biological replicate). Points indicate mean and lines standard error. **b** Principal component analysis (PCA) of normalized RNA-seq counts from developing thymocytes from XX and XY karyotype females (circle) and males (triangle) as well as a Turner syndrome patient (TS) (square). TS samples are highlighted with black arrows. **c** Average Z-score per karyotype and cell type for each cluster of dynamic gene expression throughout thymocyte development identified in Supplementary Fig. 2c. **d** Clonotype count for thymocyte subpopulations in females (F1-F4), males (M1, M3, M4) and a Turner syndrome patient (TS). **e** Occupied repertoire space of clonotypes with 1, 2-3, 4-10, 11-30, 31-100 and 101-MAX number of clones in female, male and TS samples. *P*-values (Kruskal-Wallis test with adjustment for multiple testing by Holm) are indicated at the top. **f** Diversity score in DPearly, DPlate, CD4SP and CD8SP cells of F1, F2, F3, F4, M1, M3, M4 and TS. **e**, **f** Bars indicate mean and error bars 95% CI of four thymocyte subpopulations (DPearly, DPlate, CD4SP, CD8SP) for each individual. **a**, **b**, **c**, **d**, ETP, early T cell progenitors; T-C, T cell committed thymocytes; DPearly, early double positive thymocytes; DPlate, late double positive thymocytes; CD4SP, CD4 single positive thymocytes; CD8SP, CD8 single positive thymocytes. Source data are provided as a Source Data file.

The high-fidelity maintenance of XCI observed here challenges the emerging paradigm that variable XCI is functionally relevant to T cell development in females. To directly assess the contribution of the inactive X-chromosome to human T cell development, we isolated the same six pediatric thymocyte subpopulations (Fig. 1a) from a female individual lacking a second X-chromosome (TS; 45, X0). Absence of the entire second X-chromosome was confirmed by a complete lack of common heterozygous X-linked SNPs and PCR of heterozygous repeats on the X-chromosome (Supplementary Fig. 8a, b). As expected, TS thymocytes had lower expression of escape genes compared to karyotypically normal females and lower PAR gene expression than both karyotypically normal males and females (Fig. 8a). Despite these differences, complete absence of an inactive X-chromosome had no detectable effect on T cell differentiation as measured by RNA-seq, with TS thymocytes exhibiting both an identical developmental trajectory (Fig. 8b) and subtype specific gene expression patterns as thymocytes from karyotypically normal males and females (Fig. 8c & Supplementary Fig. 8c). Thus, haploinsufficiency for immune genes (e.g., *CD99, CSF2RA, IL3RA, IL9R*) located in the PAR region has no effect on T cell development in females. Moreover, our results also suggest no effect of haploinsufficiency for gametalog escape genes; the 13 escapees with homologous genes on the Y-chromosome (e.g., *KDM6A*, *DDX3X*, *USP9X, ZFX*)^10^.

Despite the lack of a second X-chromosome TS individuals have a higher prevalence for several autoimmune diseases, similar to karyotypically normal females. A potential explanation for the heightened autoimmunity in TS females may in part be a less complex T cell receptor (TCR) repertoire due to presentation of antigens from a single copy of the X-chromosome by thymic epithelial cells. As expected, TCR rearrangement and TCR repertoire formation was apparent after the T-C stage of thymocyte development (Fig. 8d), but no clear difference between the occupied repertoire space or the diversity of the TCR repertoire of either the TS or cXCI female (F3) was observed when compared to male and female samples of normal karyotype (Fig. 8e, f).

## Discussion

An emerging hypothesis posits that aberrant or leaky expression of immuno-modulatory genes from the inactive X-chromosome in female T cells results in a hyper-responsive immune system, eliciting enhanced sensitivity to both self- and non-self-antigens. The profound importance of the imbalance in immune function has been highlighted during the COVID-19 pandemic, in which males were 3 times more likely to experience severe disease than females^3^. Understanding the molecular biology underlying this difference could result in improved sex-specific treatments for infectious and autoimmune disease.

Our serendipitous discovery of a cXCI female allowed us to create the first map of allelic expression during normal T cell development in humans and revealed that X-inactivation is highly stable throughout T cell maturation. Stability of XCI was independent of *XIST* levels and resulted in minimal sex-biased expression of X-linked genes in thymocytes. Together these findings motivate a refinement of the attractive but underdeveloped paradigm of atypical escape from XCI of immune genes during T cell development as a key driver in the altered adaptive immune response between the sexes. However, as our results are based exclusively on pediatric thymi, they do not exclude the occurrence of atypical escape during (i) thymocyte development later in life, when most sex-biased autoimmune diseases such as SLE are diagnosed or (ii) during the process of T cell activation/differentiation in which atypical XCI has also been observed^13^. Further, given the limited sample size (*N*_MALE_ = 3, *N*_FEMALE_ = 4), our study cannot account for the potential effect of inter-group genetic variation as a confounder of the observed sex differences.

While our results appear to exclude a general relaxation of XCI and associated increase of gene expression across the inactive X-chromosome during T cell development, they do not exclude localized loss of XCI resulting in biallelic expression of specific X-linked genes. Whereas the use of samples from a cXCI female allowed direct determination of XCI status, this could only be achieved for the 127 X-linked genes containing heterozygous SNPs in this female. Indeed, the 127 genes assayed did not include genes such as *TLR7* which is the most frequently reported atypical escapee in human immune cells^12,13,33^. Interestingly, most studies reporting atypical XCI maintenance in lymphocytes have used RNA-FISH to detect primary transcripts in situ^12,13,33^, whereas here we assayed polyA mRNA. Taken together, it is tempting to speculate that post-transcriptional regulation of atypically expressed X-linked pre-RNAs could restrict their processing into mature polyA RNA. Indeed, X-linked transcripts have RNA modification profiles distinct to those of autosomal transcripts which have been suggested to alter X-linked RNA stability and turnover^37,38^.

Finally, these data represent the first high-resolution map of X-inactivation during any normal developmental process in humans and are a unique resource for investigating the biology of XCI in human health and disease.

## Methods

### Ethics statement

The collection of human samples was approved by the Swedish Ethical Review Authority (Dnr 2022-07166-02 & Dnr 217-12, 2012-04-27) and all parents gave written informed consent for participation in the study. The study was performed in accordance with the declaration of Helsinki.

### Study participants

Thymic lymphocytes were isolated from nine participants. Four XX-females, four XY-males and one X0 Turner syndrome patient. Participants had a mean age of 15 months (2–38 months). Information on inclusion of each participant in individual experiments can be found in Supplementary Table 1.

### Isolation of thymic lymphocytes and PBMCs

The thymic tissue was placed in cold PBS immediately after surgical removal and tissue processing started within one hour. Thymic lymphocytes were isolated from tissue by mechanical disassociation and filtration. The thymic single cell suspension and peripheral blood were density centrifuged on Ficoll-Paque Plus (Cytiva, 17144003).

### Sorting of thymocyte populations

The enrichment of CD34+ cells was performed with DynabeadsTM CD34 Positive Isolation Kit (Invitrogen, 11301D). Enriched and non-enriched samples were then stained with antibodies listed in Supplementary Table 4. Live/Dead - FVD506 (eBioscience, 65-0866-14) was used as viability marker. The biotinylated antibody was stained with Brilliant Violet 421 Streptavidin (Biolegend, 405226). Thymocyte cell populations were sorted on a SH800 cell sorter (Sony) or FACSAria Fusion (Beckton Dickinson) following the gating strategy described in Supplementary Fig. 1.

### DNA and RNA extraction

Genomic DNA and RNA were extracted using the Quick-DNA/RNA Miniprep Kit (Zymo Research, D7001). RNA samples were treated with DNase I (5U; Zymo Research, E1010) in column to digest DNA. Similarly, DNA was treated using RNase A (Thermo Fisher, EN0531). DNA was quantified using the NanoDrop 2000 Spectrophotometer (Thermo Fisher). RNA was quantified and quality was assessed with 2100 Bioanalyzer (Agilent, 5067-1511). All samples had a RIN score ≥ 8.

### RNA-seq library preparation

100 ng RNA from thymocyte populations was diluted in nuclease-free water to a total volume of 50 µl and enriched for mRNA using the NEBNext Poly(A) mRNA Magnetic Isolation Module based on Oligo-dT-beads (New England Biolabs, E7490S). For the Turner syndrome patient (TS) sequencing libraries were prepped from only 50 ng of RNA. Fragmentation, cDNA synthesis, dA-tailing, adapter ligation, indexing and PCR enrichment for 11 cycles was done using the NEBNext Ultra II RNA Library Prep Kit for Illumina (New England Biolabs, E7770S) and NEBNext Multiplex Oligos for Illumina Index Primers Set 1 and Set 2 (New England Biolabs, E7335S and E7500S) following the manufacturer’s instructions. Library size distribution and quality was checked using 2100 Bioanalyzer (Agilent, 5067-4626). Library concentration was determined on the Quantus or Qubit Fluorometer with QuantiFluor ONEdsDNA Dye (Promega, E4871) or Qubit 1X dsDNA HS Assay (Thermo Fisher, Q33231), respectively. Libraries were pooled and diluted to a concentration of 800 pM and sequenced on the NextSeq 2000 machine using the NextSeq 1000/2000 v3 Reagents (Illumina, 20046812 or 20040560) with paired-end reads of 100 bases each and 30–68 million reads per sample.

### Differential expression of thymocyte RNA-seq data

Transcriptome mapping was performed using Salmon^39^ (v1.10.2) (–gcBias –seqBias –numBootstraps 100) using known Refseq transcripts (NM_* and NR_*, excluding all Y-PAR transcripts) from the GRCh38.p12 assembly (GCF_000001405.38_GRCh38.p12_rna.fna.gz) as a reference. Library type options were Thymocyte: IU. Raw fastq reads were quality trimmed using FastP^40,41^ (v.0.23.4) with default settings. Abundance estimates were converted to h5 format using Wasabi (https://github.com/COMBINE-lab/wasabi) and data normalization and differential expression analysis was performed using Likelihood Ratio test (LRT) tests and gene-level p-value aggregation as implemented in Sleuth^42,43^. Normalized transcript abundances were aggregated to gene-level abundances using tximport^44^. Genes with mean TPM (across all thymocyte subtypes) of less than 1 were filtered out. Temporal clustering was performed using k-means clustering in R where seven clusters were the most informative. Gene set enrichment analysis was performed using GSEA (v4.3.3)^45^ on pre-ranked lists using default settings and the GO biological processes database. GO enrichment analysis was performed using PANTHER^46^ overrepresentation test (release 20240226) and the GO biological processes database. Transitional genes were identified by contrasting T-C and DPearly, DPlate and CD4SP, DPlate and CD8SP as well as CD4SP and CD8SP using Wald test (WT) and gene-level p-value aggregation as implemented in Sleuth^42,43^. The 20 genes with the largest difference in TPM (10 genes with higher TPM in either thymocyte subtype) for each individual contrast and with a *P*-value < 1 × 10^−3^ were selected.

### Analysis of sex-biased gene expression in RNA-seq data

To determine sex-biased gene expression throughout thymocyte development, raw fastq reads were quality trimmed with FastP (v.0.23.4) using default settings. Transcriptome mapping was performed using Salmon (v1.10.2) (–gcBias –seqBias –numBootstraps 100) using known Refseq transcripts (NM_* and NR_*, excluding all Y-PAR transcripts) from the GRCh38.p12 assembly (GCF_000001405.38_GRCh38.p12_rna.fna.gz) as a reference. Abundance estimates were converted to h5 format using Wasabi (v1.0.1, https://github.com/COMBINE-lab/wasabi) and data normalization and male vs female log2-fold change (FC (log2)) was calculated using WT tests and gene-level *p*-value aggregation as implemented in Sleuth. When creating the sleuth object, transformation_function = function(x) log2(x + 0.5) was used to ensure that the WT test output log changes as log2. Normalized transcript abundances were aggregated to gene-level abundances using tximport^44^. Genes with a mean TPM (across all thymocyte subtypes) of less than 1 were filtered out. Genes with a *P*-value < 1 × 10^−5^ and an absolute FC (log2) > 0.2 were classified as sex-biased. To investigate sex-biased gene expression across multiple tissues, male and female samples for 47 tissues were downloaded from GTEx, aiming for 10 of each sex (see Supplementary Table 3 for complete list of tissues and sample counts). Briefly, data was downloaded from AnVIL using the Gen3 client (v1.0.0) as aligned BAM files, which were sorted and converted into fastq files using Samtools (v1.7)^47^ and finally processed through the pipeline described above but utilizing FastP (v.0.21.0) and Salmon (v0.7.2) instead. Genes with a mean TPM (calculated for each tissue individually) of less than 1 were excluded.

### Isolation of primary immune cells

Peripheral blood mononuclear cells (PBMCs) were extracted from buffy coats using Lymphoprep (Axis Shield Poc As, 1114545). 35 ml blood was layered on 15 ml Lymphoprep and centrifuged at 800 × *g* for 30 minutes. The interphase containing PBMCs was transferred to a fresh falcon tube for DNA and RNA extraction or for isolation of CD4+ and CD8+ T cells. Naïve T cells were isolated from PBMCs with the Naive CD4+ T Cell Isolation Kit II (Miltenyi Biotec, 130-094-131) and the QuadroMACS Separator (LS Columns, Miltenyi Biotec, 130-042-401) as instructed by the manufacturer. CD4+ T cells were similarly isolated by magnetic bead separation using the CD4+ T Cell Isolation Kit (Miltenyi Biotec, 130-096-533). The CD4 negative fraction was further enriched for CD3+ and CD8+ cells by antibody staining and FACS sorting (Anti-CD3-Pacific Blue, clone UCHT1, BioLegend, 300418 and Anti-CD8-APC, clone RPA-T8, BD, 555369).

### qPCR

Using qPCR, expression of selected genes on chromosome X was analyzed in primary immune cells, commercially available RNA samples from bone marrow (biochain, ATR1234024; Zyagen, HR-704; TaKaRa, 63659) and a panel of 15 further tissues (Ambion, AM6000). RNA was dsDNase I treated (Thermo Fisher, EN0771, 37 °C for 2 minutes) and converted to cDNA (High Capacity cDNA Reverse Transcription Kit, Applied Biosystems, 4368814, 37 °C for 2 hours, 85 °C for 5 minutes). Following, qPCR was carried out using Fast Universal PCR Master Mix (Thermo Fisher Scientific, 4352042) and TaqMan probes (Supplementary Table 5) on the QuantStudio 7 Real-Time PCR Systems (Thermo Fisher) for 40 cycles. Expression was analyzed relative to *GAPDH* expression (ΔCt).

### Alignment method comparison

To compare how alignment method affects sex-bias quantification, GTEx RNA-seq data for 9 tissues was downloaded (see Supplementary Table 3 for complete list of tissues and sample counts) and aligned using either pseudo- or reference-based alignment. Briefly, data was downloaded from AnVIL using the Gen3 client as aligned BAM files, which were sorted and converted into BAM files with Samtools (v1.7). Raw fastq reads were quality trimmed with FastP (v.0.21.0) using default settings. For pseudo-alignment, the same pipeline as described under *Analysis of sex-biased gene expression in RNA-seq data* was used. For reference-based alignments, quality trimmed fastq reads were aligned with STAR 2-pass mode (v.2.7.6a)^48^ using an index built with GCA_000001405.15_GRCh38_no_alt_analysis_set.fna as the reference genome and –sjdbGTFfile gencode.v35.annotation.gtf. Following alignment, BAM files were sorted with Samtools (v1.7) and deduplicated with Sambamba (v0.7.1)^49^. To count reads featureCounts (v2.0.3)^50^ was used, specifying -p –countReadPairs -t exon -g gene_id -a gencode.v35.annotation.gtf. To obtain male vs female log2-fold change, edgeR (v. 3.28.1)^51^ glmQLFit was used. Genes with a mean transcript per million (TPM) < 1 were excluded.

### Analysis of sex-biased gene expression in Affymetrix data

Pre-processed affymetrix data was obtained from GTEx and downloaded from GEO under accession number GSE45878. Log2 fold-change was calculated by using the following formula: log2(mean(female expression) / mean(male expression)).

### HUMARA assay

200 ng of DNA from thymic lymphocytes and peripheral blood mononuclear cell (for females F1-3) or DPearly cells (for female F4) was incubated with the methyl-sensitive restriction enzyme *Hpa*II (New England Biolabs, R0171; 10 U per reaction) in a total volume of 10 µl for 16 hours at 37 °C. A negative sample without *Hpa*II was included for each DNA sample in all steps. Following, the restriction enzyme was heat inactivated by incubation at 80 °C for 20 minutes. 5 µl of digested and undigested DNA (equivalent to 100 ng DNA) was used in a PCR reaction for the CAG repeat in exon 1 of the AR gene on chromosome X (Supplementary Table 6). The forward primer was labeled with FAM while the reverse primer remained unlabeled. The PCR reaction had a total volume of 50 µl and included Phusion High-Fidelity DNA Polymerase (New England Biolabs, M0530; 1 U per reaction), 200 µM dNTPs and 500 nM of each primer. PCR cycling was as follows: initial denaturation at 98 °C for 30 seconds, followed by 27 cycles of 98 °C for 10 seconds, 62 °C for 30 seconds and 72 °C for 30 seconds; and a final extension at 72 °C for 5 minutes. 1 µl of the PCR fragments was size separated on a 3500 Genetic Analyzer (Applied Biosystems) using Hi-Di staining (Applied Biosystems, 4311320) and a LIZ 600 size standard (Applied Biosystems, 4366589). Analysis was done using the seqinr package^52^.

### Whole exome sequencing

250 ng DNA from peripheral blood mononuclear cell (females F1-3) or DPearly cell (female F4) was diluted in 1X Tris-EDTA (Invitrogen, 12090015) to a volume of 130 µl and sonicated in the Covaris S220 to a fragment size of 200 – 300 basepairs. After sonication, fragmented DNA was concentrated to 50 µl in 1X Tris-EDTA with the DNA Clean & Concentrator kit (Zymo research, D4013) following the manufacturer’s instructions. Whole exome sequencing libraries were prepared with the SureSelect XT HS Reagent Kit for Illumina (ILM) platform (Agilent, G9702A) and target regions were enriched using probes for exons and UTRs (SureSelectXT Human All Exon V6 + UTR; Agilent, 5190-8881). The manufacturer’s instructions were followed with adapter-ligated genomic DNA libraries PCR amplified for 8 cycles before 1 µg of these libraries was used for exome+UTR capture. Enriched libraries were PCR amplified for 9 cycles. Library quality and size distribution was assessed with the TapeStation D1000 ScreenTape (Agilent, 5067-5582) and quantification was done on the Qubit Fluorometer with the 1X dsDNA HS Assay kit (Thermo Fisher, Q33231). Libraries were pooled and diluted to a concentration of 800 pM and sequenced on the NextSeq 2000 machine (NextSeq 1000/2000 P2 Reagents (300 Cycles) v3; Illumina, 20046813) with paired-end reads of 150 bp each and > 124 million read-pairs per sample.

### Allele-specific expression analysis

For single nucleotide polymorphism (SNP) calling in thymocyte samples, whole exome sequencing (WES) raw fastq reads were quality trimmed using FastP^40,41^ (v.0.23.4) with default settings. BWA-MEM^53^ (v0.7.18) was used for mapping reads to the human genome build 38 (hg38, GCA_000001405.15_GRCh38_no_alt_analysis_set.fna) using default settings. Aligned reads were processed through the GATK^54^ best practice germline short variant discovery pipeline, using base quality score recalibration and local realignment at known indels (GATK v.4.5.0.0.). Indels (insertions and deletions) and SNPs were jointly called across all 4 samples. Default filters were applied to indel and SNP calls using the variant quality score recalibration (VQSR) approach of GATK. The thymocyte subpopulation RNA-seq raw fastq reads were quality trimmed using FastP (v.0.23.4) using default settings. RNA-seq reads were aligned with STAR 2-pass mode (v.2.7.11b) using an index built with GCA_000001405.15_GRCh38_no_alt_analysis_set.fna as the reference genome and –sjdbGTFfile gencode.v43.annotation.gtf. To reduce the issue of reference bias^55^, WASP filtering was performed. Briefly, heterozygous variants that passed VQSR filtering were extracted for each sample from the WES VCFs using GATK SelectVariants, and subsequently passed to STAR via –varVCFfile and–waspOutputMode. Reads that did not pass the WASP filtering were filtered out using bamtools^56^ (v2.5.1). Following data pre-processing, allele-specific expression (ASE) analysis of the allele counts for biallelic heterozygous variants were retrieved from RNA-seq data using GATK ASEReadCounter (v.4.5.0.0). Heterozygous variants that passed VQSR filtering were first extracted for each sample from WES VCFs using GATK SelectVariants. Sample-specific VCFs and RNA-seq BAMs were inputted to ASEReadCounter requiring minimum base quality (phred) of 20 in the RNA-seq data. For downstream processing of the ASE data, we applied further filters to the data to conservatively remove potentially spurious sites, requiring a variant call read depth of ≥20 reads per allele, excluding any variants in which the minor allele was less than 10% of the total read count and requiring an RNA-seq read depth of ≥10. In case of multiple ASE sites in a gene, only the site with the highest RNA-seq read count was included. When analyzing the minor allele read count a threshold of RNA-seq total read count ≥ 10 & WES total read count >20 was used to include more hSNPs but still only keep high-confidence calls. When investigating minor allele read counts, the allele with the fewest reads was assigned minor allele. For investigating the minor allele count of immune genes, a list of 86 immune genes was compiled from the literature^28–30^.

### Illumina EPIC methylation array

250 ng of DNA from each thymocyte population from females F1, F3 and F4 was diluted in nuclease-free water and the Infinium MethylationEPIC BeadChip v1 (Illumina, WG-317) was run on the NextSeq 550 sequencing machine. In total three chips were processed at the same time with all samples from each female run on the same chip, resulting in six samples per chip (all six thymocyte populations for one female).

### Thymocyte Illumina EPIC methylation array analysis

Genome-wide methylation profiling of normal human thymocyte DNA was performed using Illumina EPIC DNA methylation arrays. Raw data was pre-processed using the default parameters of the Chip Analysis Methylation Pipeline (ChAMP)^57^. The data was normalized using Beta-Mixture Quantile (BMIQ) normalization^58^. Hierarchical clustering was performed using pvclust^59^ and 10,000 bootstraps.

### X-inactivation status categorization

Genes had their XCI status classified either by using a previous assessment^10^ or by performing our own classification. Briefly, genes were assigned as high-confidence escapees if either a) allele-specific expression was lower or equal to 0.4 or b) if the gene’s mean methylation −500 TSS value was below 0.25 and sex-biased expression above 0.2 log2FC. Genes with a mean methylation −500 TSS value below 0.25 and sex-biased expression above 0 but below 0.2 log2FC were assigned low-confidence escapees. Genes were assigned as inactive genes if a) allele-specific expression was higher than 0.4 or b) if the genes mean methylation −500 TSS value was above 0.25 and its sex-biased expression was below 0.2 log2FC. Genes that fell outside of these criteria were assigned “unclassified” status. This category thus includes genes that lack ASE coverage and either have a methylation value below 0.25 and a sex-bias below 0 log2FC or a methylation value above 0.25 but with a sex-bias above 0 log2FC.

### X-chromosome repeat amplification

The polymorphic AR and RP2 repeats on the X-chromosome were amplified by PCR using FAM-labeled forward and unlabeled reverse primers (Supplementary Table 6). The PCR reaction had a total volume of 20 µl including 100 ng DNA from thymic lymphocytes of females F3-4 and the Turner syndrome patient (TS), Phusion High-Fidelity DNA Polymerase (0.4U per reaction; New England Biolabs, M0530), 400 µM dNTPs, 3% v/v DMSO, 400 nM of each AR-primer and 300 nM of each RP2-primer. PCR cycling was as follows: initial denaturation at 98 °C for 30 seconds followed by 30 cycles of 98 °C for 10 seconds, 63 °C for 30 seconds and 72 °C for 30 seconds. Finally, a final extension at 72 °C for 5 minutes was performed. The PCR product was diluted 1:10 in nuclease-free water and 1 µl of the diluted PCR fragments were size separated on a 3500 Genetic Analyzer (Applied Biosystems) using Hi-Di staining (Applied Biosystems, 4311320) and LIZ 600 size standard (Applied Biosystems, 4366589). The resulting electropherograms were visualized using the seqinr package^52^.

### TCR repertoire analysis

To characterize the T cell receptor (TCR) repertoires in the thymocyte bulk RNA-seq data, the TRUST4 (v1.1.1)^60^ software was used. TRUST4 performs de novo assembly, annotation, and consensus assemblies of TCRs. Briefly, raw fastq reads were quality trimmed using FastP (v.0.23.4) using default settings and subsequently run through TRUST4 using default settings and provided reference files. The TRUST4 output was then analyzed with Immunarch (10.5281/zenodo.3367200). Prior to TCR analysis, data was downsampled with the repSample function to account for difference in sequencing depth. ETP and T-C cells were excluded from diversity score calculations given their inherently low diversity score. A Kruskal-Wallis test, correcting for multiple testing with the Holm method, was used to test for statistical significance in occupied repertoire space between the samples (Fig. 8e).

### Smart-seq2 on sorted thymocyte populations

For one female individual (F4) we conducted single cell sequencing using the Smart-seq2 approach as described by Picelli et al.^61,62^. In brief, six thymocyte subpopulations were single cell sorted into 96-well PCR plates, directly into 4 µl lysis buffer (0.05% Triton X-100, 2.5 µM oligo-dT primers, 2.5 mM dNTPs, 2.5 mM RNaseOUT (Thermo Fisher, 10777019)). After denaturation of RNA at 72 °C for 3 minutes, 5.5 µl cDNA synthesis buffer was added to each well (100U SuperScrip II Reverse Transcriptase (Thermo Fisher, 18064071), 9 mM DTT, 1.5 M Betaine, 18 mM MgCl_2_, 1.8 µM LNA strand-switching primer, 4.5 mM RNaseOUT) and incubated at 42 °C for 90 minutes followed by 10 to 12 cycles (dependent on cell type) of 50 °C for 2 minutes and 42 °C for 2 minutes before a final incubation at 70 °C for 15 minutes. The resulting cDNA (10 µl) was amplified in a total volume of 25 µl using the KAPA HiFi HotStart ReadyMix PCR Kit (Roche, KK2602) for 22 to 24 cycles (dependent on cell type). After bead purification with AMPure XP beads (Beckman Colter, A63880) cDNA was quantified and quality was assessed on the Bioanalyzer (Agilent, 5067-4626). An average of 1.5 ng cDNA was tagmented using the Tn5 transposase in a total volume of 20 µl including 10 mM Tris-HCl, 5 mM MgCl_2_ and 10% Dimethylformamide for 10 minutes at 55 °C. The reaction was terminated by addition of 3.5 µl 0.2% Sodium dodecyl sulfate. Finally, sequencing indexes were attached and libraries were PCR amplified for 15 cycles using the KAPA HiFi PCR Kit (1U per reaction; Roche, KK2102). All single cell sequencing libraries of the same cell type were pooled and quantified before libraries were pooled and diluted further for sequencing on the NextSeq 2000 platform (150 bp paired-end, median number of raw reads per cell 0.87 M for all cell types).

### Analysis of Smart-seq2

For the allele-specific expression analysis using Smart-seq2, the data was processed exactly like the bulk ASE data, however allowing several hetSNPs per gene, given that they pass the read count thresholds (see *Allele-specific expression analysis* for details). Clustering the cells into clusters was performed by first identifying cells with detected read counts for *XIST* and leveraging that allelic information to cluster other cells using other genes than *XIST*. Briefly, cells with *XIST* read counts were assigned into Cluster 1 or cluster 2 based on which allele was detected. Based on that clustering, inactive genes with a consistent monoallelic expression per cluster (*ATRX*, *BEX4*, *ITM2A*, *LAMP2*, *MORF4L2*, *PIN4* and *TMSB4X*) were used to assign cells into clusters. Clustering results for each cell and which gene was used for the assignment can be found in Supplementary Data 9. Genes with a mean reference ratio between 0.1 and 0.9 (across all cells per cluster) were classified as escaping from XCI. This can be achieved in two ways, either by a) both reference and alternative allele being identified within the same cell or b) both reference and alternative allele being detected within the same cluster. hetSNPs were required to be detected in more than one cell. For gene expression quantification and UMAP generation, STAR and Seurat^63,64^ (v5.1.0) was utilized. Briefly, fastq reads were quality trimmed using FastP (v.0.23.4) using default settings. STAR (v.2.7.11b) was run with the following options: –soloType SmartSeq –soloUMIdedup Exact –soloStrand Unstranded –limitOutSJcollapsed 10000000 –soloCellFilter None, using GCA_000001405.15_GRCh38_no_alt_analysis_set.fna and gencode.v43.annotation.gtf for index generation. Downstream analysis of the data was performed using Seurat, excluding cells with a mitochondrial gene count >10%, cells with a higher ribosomal content than 20% and requiring a read count between 125k and 1 M per cell. Briefly, data was normalized using the SCTransform function (vst.flavor = v2), variable features identified with FindVariableFeatures (selection.method = “vst”, nfeatures = 2000), followed by ScaleData (default settings), RunPCA (npcs = 30), FindNeighbors (dims = 1:10) and finally runUMAP (dims = 1:10, n_neighbors = 30).

### Reporting summary

Further information on research design is available in the Nature Portfolio Reporting Summary linked to this article.

## Supplementary information

Supplementary Information

Description of Additional Supplementary Files

Supplementary Data 1

Supplementary Data 2

Supplementary Data 3

Supplementary Data 4

Supplementary Data 5

Supplementary Data 6

Supplementary Data 7

Supplementary Data 8

Supplementary Data 9

Supplementary Data 10

Supplementary Data 11

Reporting Summary

## Source data

Source Data

## Data Availability

All sequencing data generated in this study (including bulk and single-cell RNA-seq, whole exome sequencing and EPIC array data) have been deposited in the Swedish National Data Service (SND, https://snd.gu.se/, a data repository certified by Core Trust Seal) under accession code 2022-112-1 [10.5878/ayae-p143]. Access to restricted data will require completion of a Data Access Request (DAR) via the SND website. Each Data Access Request will be evaluated individually according to Swedish legislation. Source data are provided with this paper.

## Code availability

All code to reproduce figures is available at 10.5281/zenodo.13933086.
