# Supplementary material for: A landscape of X-inactivation during human T cell development
Source: Nat Commun. 2024 Dec 4;15:10527. doi: 10.1038/s41467-024-54110-7 (PMC11618795; doi:10.1038/s41467-024-54110-7)
Supplement: Supplementary file 1 — Supplementary Information [file 41467_2024_54110_MOESM1_ESM.pdf]

Supplementary Information

A landscape of X-inactivation during human T cell development

Authors: Björn Gylemo[1,*], Maike Bensberg[1,*], Viktoria Hennings[2,3,*], Christina Lundqvist[2,*], Alessandro Camponeschi[2], Dóra Goldmann[1], Huan Zhang[1], Aida Selimović[1], Antonio Lentini[1,†], Olov Ekwall[2,3, †], Colm E. Nestor[1, †]

Affiliations:

[1]Crown Princess Victoria Children´s Hospital, and Department of Biomedical and Clinical Sciences (BKV), Linköping University, Linköping, Sweden.

[2]Department of Rheumatology and Inflammation Research, Institute of Medicine, The Sahlgrenska Academy, University of Gothenburg, Gothenburg, Sweden.

[3]Department of Pediatrics, Institute of Clinical Sciences, The Sahlgrenska Academy, University of Gothenburg, Gothenburg, Sweden.

[*]These authors contributed equally: Björn Gylemo, Maike Bensberg, Viktoria Hennings, Christina Lundqvist

[†] These authors jointly supervised this work: Antonio Lentini, Olov Ekwall, Colm E. Nestor

Correspondence to: colm.nestor@liu.se

Table of contents:

Supplementary Figures 1 – 8

Supplementary Tables 1 – 6

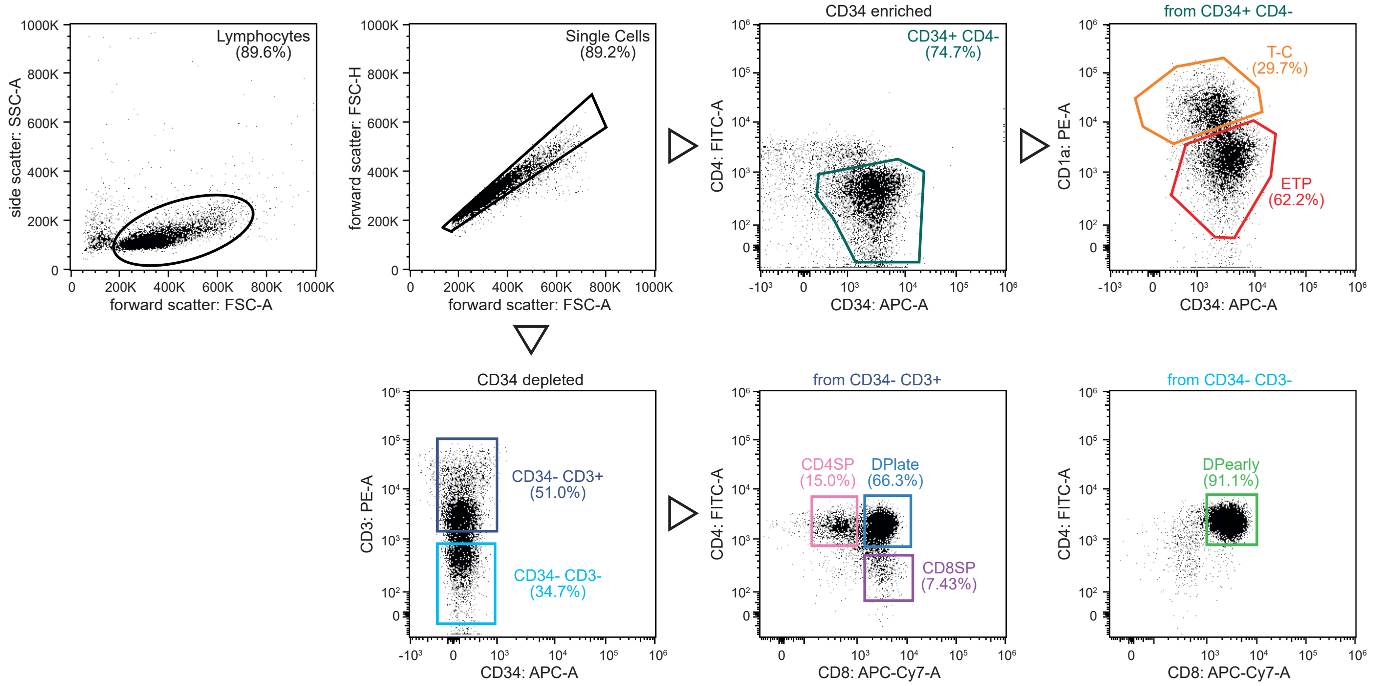

Supplementary Figure 1: Thymocyte sorting. FACS sorting strategy for isolation of primary human thymocytes. ETP, early T-cell progenitors (CD34+, CD1a-, CD4-); T-C, T-cell committed thymocytes (CD34+, CD1a+, CD4-); DPearly, early double positive thymocytes (CD34-, CD3-, CD4+, CD8+); DPlate, late double positive thymocytes (CD34-, CD3+, CD4+, CD8+); CD4SP, CD4 single positive thymocytes (CD34-, CD3+, CD4+, CD8-); CD8SP, CD8 single positive thymocytes (CD34-, CD3+, CD4-, CD8 +). Antibodies used are listed in Supplementary Table 4. Source data are provided as a Source Data file.

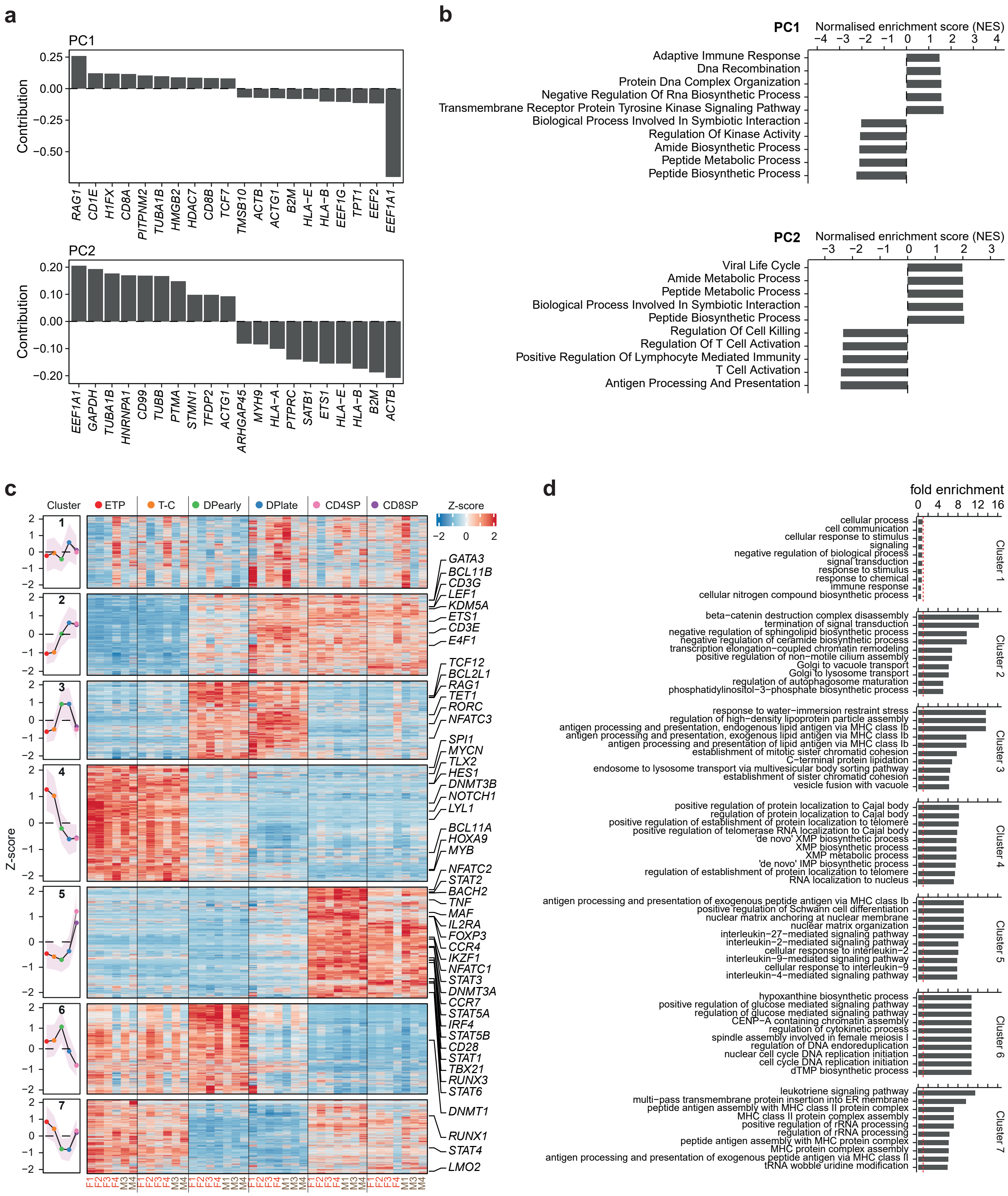

Supplementary Figure 2: Expression dynamics during human thymocyte development. a, Top 20 genes contributing to principal component 1 (PC1) (top) and PC2 (bottom) in Fig. 1b. **b**, Normalized enrichment score of gene set enrichment analysis on PC1 and PC2 in Fig. 1b. **c**, Expression heatmap of 7 temporal gene clusters that show dynamic expression throughout thymocyte development. **d**, Panther GO enrichment analysis on the temporal clusters in panel **c**. n = 6 biological replicates for each thymocyte subpopulation (3 females and 3 males). Source data are provided as a Source Data file.

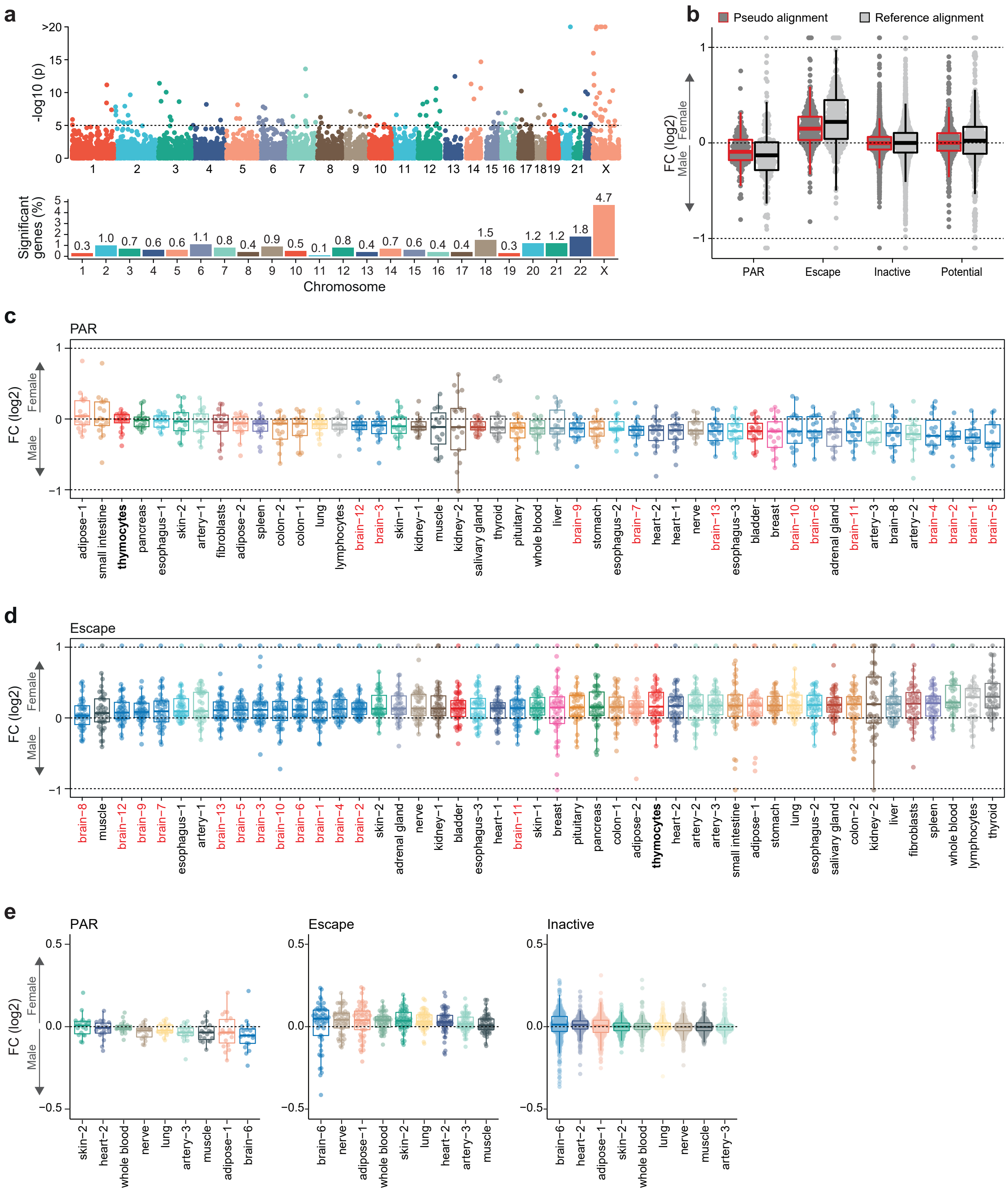

Supplementary Figure 3: Sex biased expression in human primary tissues. a, Significance values (-log10(P)) from a two-tailed Wald test of sex-biased gene expression across all expressed genes and all chromosomes except chromosome Y (top) and proportion of significant sex-biased genes for each chromosome except chromosome Y (bottom). Combination of six thymocyte subpopulations for n = 6 biological replicates (3 females and 3 males). **b**, Sex-biased expression as log2 of fold change (FC) female over male of gene expression in PAR (pseudoautosomal region), escape, inactive and potential escape genes when aligned using a pseudo alignment or a reference alignment approach. See methods for details. **c,d**, PAR (**c**) and escape (**d**) gene expression sex-bias in 47 tissues from GTEx. **e**, Sex-bias across PAR, escape and inactive genes in Affymetrix GTEx data. **b,c,d,e**, Boxplot representing median (central line),

first and third quartiles (Q1 and Q3, respectively) (box edges) and 1.5*inter quartile range (IQR) from Q1 and Q3 (whiskers). **c,d,e** n = 20 (10 male and 10 female for each tissue). See Supplementary Table 3 for a complete list of tissue abbreviations and sample counts. Source data are provided as a Source Data file.

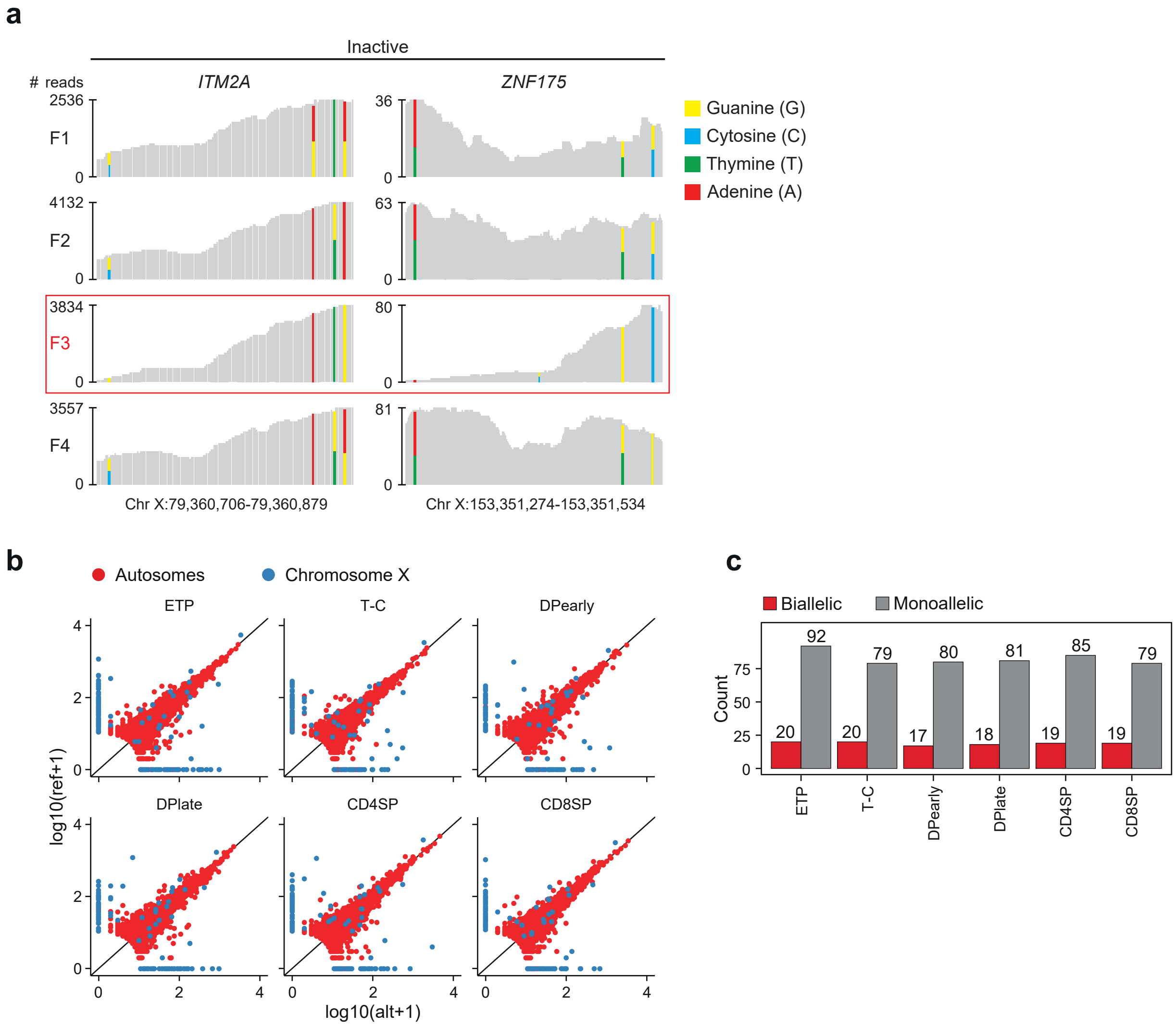

Supplementary Figure 4: Identification of a female with completely skewed XCI. a, RNA-seq read counts of inactive X-chromosome genes ITM2A and ZNF175 in females F1-F4. Heterozygous single nucleotide polymorphisms are indicated as colored bars (G: yellow, C: blue, T: green, A: red). **b**, Allele-specific expression (ASE) of genes on autosomes (red) and on the X-chromosome (blue) in thymocyte subpopulations in F3. **c**, Number of biallelic and monoallelic genes on chromosome X per thymocyte subpopulation in F3. Source data are provided as a Source Data file.

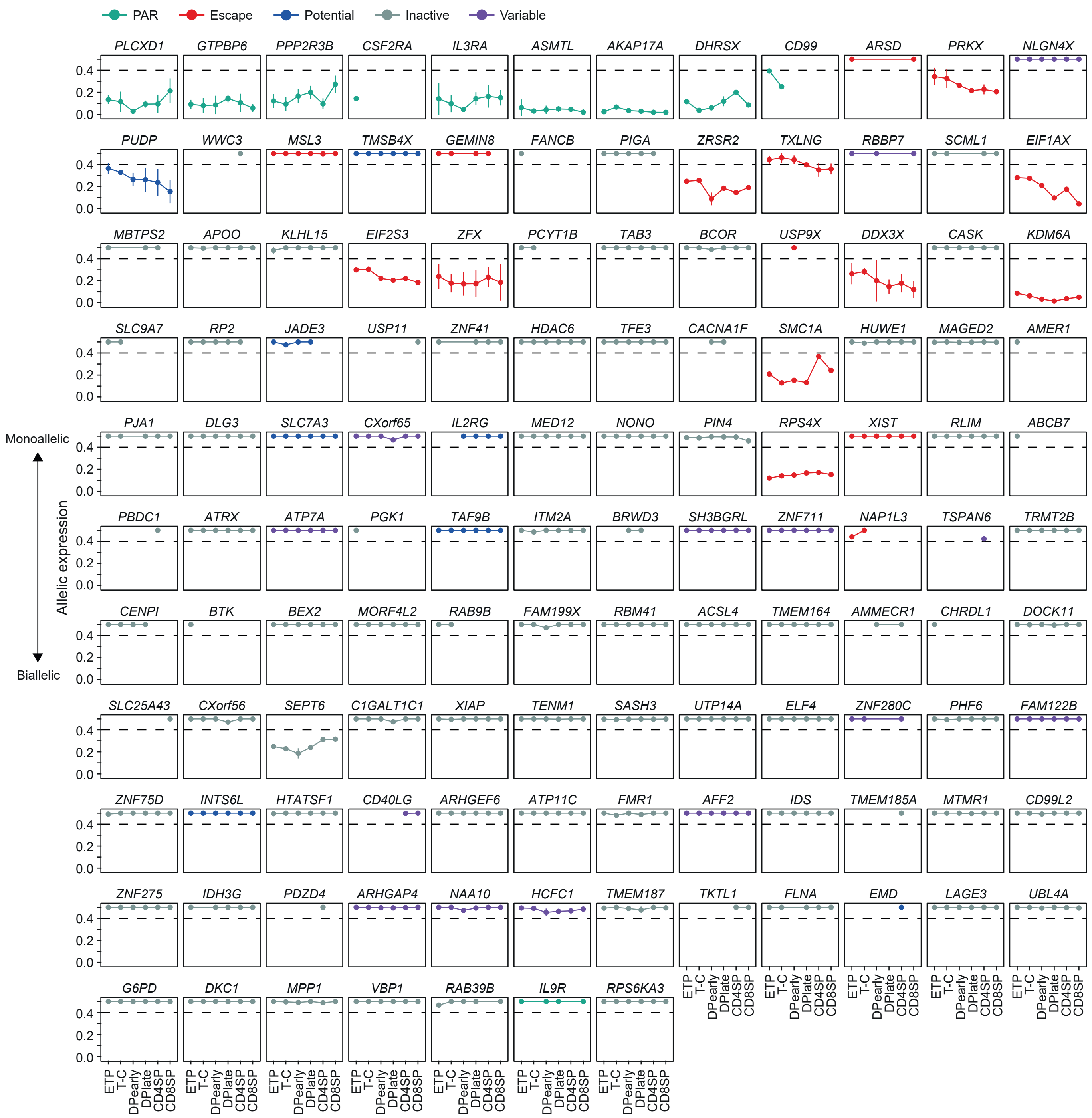

Supplementary Figure 5: Map of escape from X-inactivation. Allele-specific expression of all covered X-linked genes across thymocyte development in female 3 (F3). XCI status defined based on previous assessment with potential XCI escape genes (blue) previously not investigated or classified as unknown. Dot and lines indicate mean and standard deviation, respectively. Source data are provided as a Source Data file.

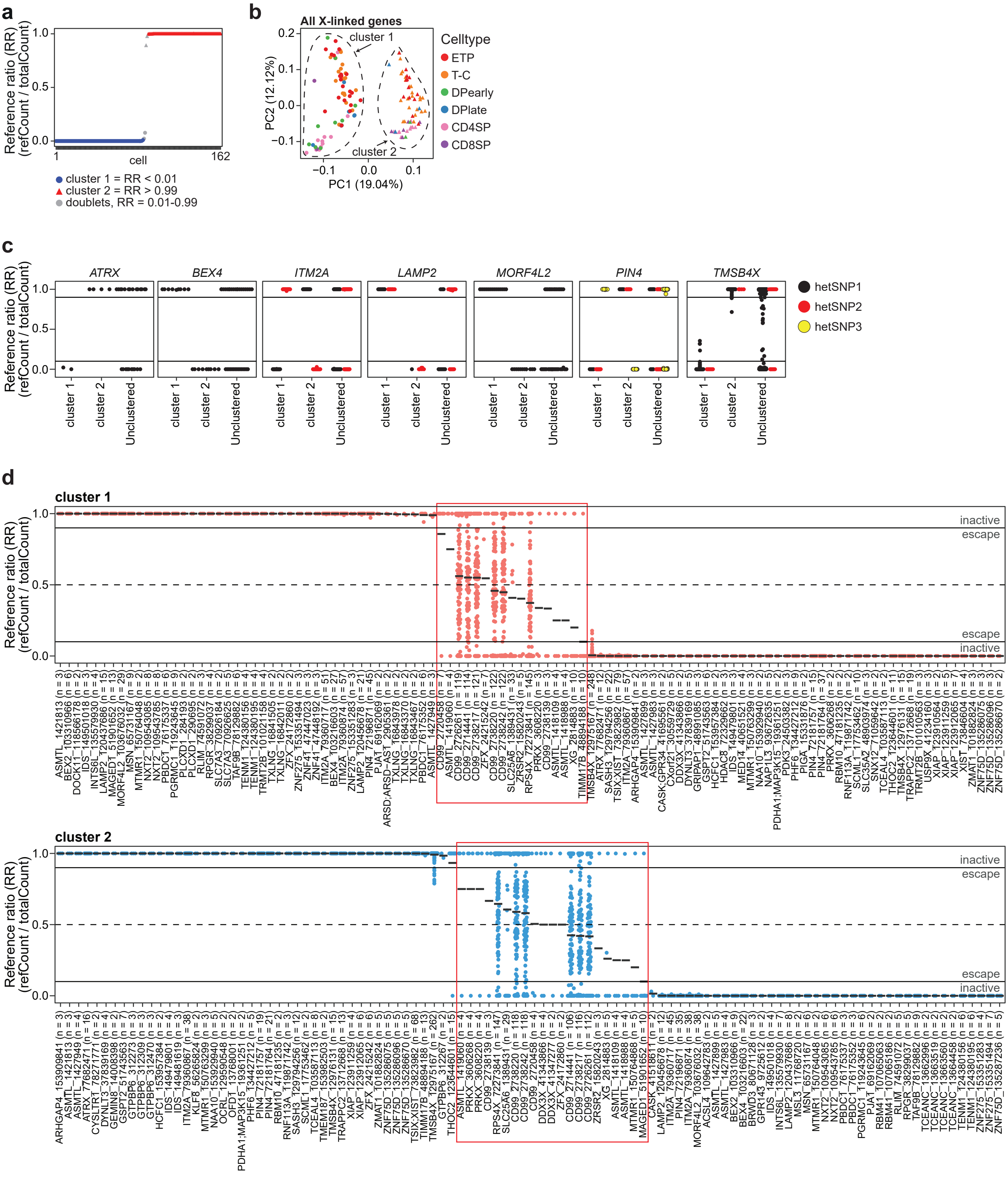

Supplementary Figure 6: Single cell sequencing of primary human thymocyte populations. Single cell sequencing (Smart-seq2) for sorted thymocyte subpopulations from one female sample, F4. **a**, Reference ratio (RR) (reference count / total read count) of XIST per cell. Cells with XIST RR < 0.01 were assigned to cluster 1, cells with an XIST RR > 0.99 assigned to cluster 2. Cells with an XIST RR between 0.01 and 0.09 were classified as doublets. **b**, Principal component (PC) analysis on the RR of all X-linked genes with detected XIST allele-specific expression. ETP, early T-cell progenitors; T-C, T-cell committed thymocytes; DPearly, early double positive thymocytes; DPlate, late double positive thymocytes; CD4SP, CD4 single positive thymocytes; CD8SP, CD8 single positive thymocytes **c**, RR of genes with a consistent allelic expression per cell assigned into clusters based on panel **a,b** (cluster 1 or cluster 2) and cells without detected XIST allele-specific expression (unclassified). Genes with several heterozygous SNPs

colored in black, red or yellow. **d**, RR from single cells pooled based on clusters identified in panels. Black lines indicate mean RR per gene and cluster. **a,b,c**. Escape genes (reference ratio: 0.1 – 0.9) highlighted in red box. n, number of cells expressing the SNP. Numbers after gene symbols are the genomic position of the heterozygous SNP. Source data are provided as a Source Data file.

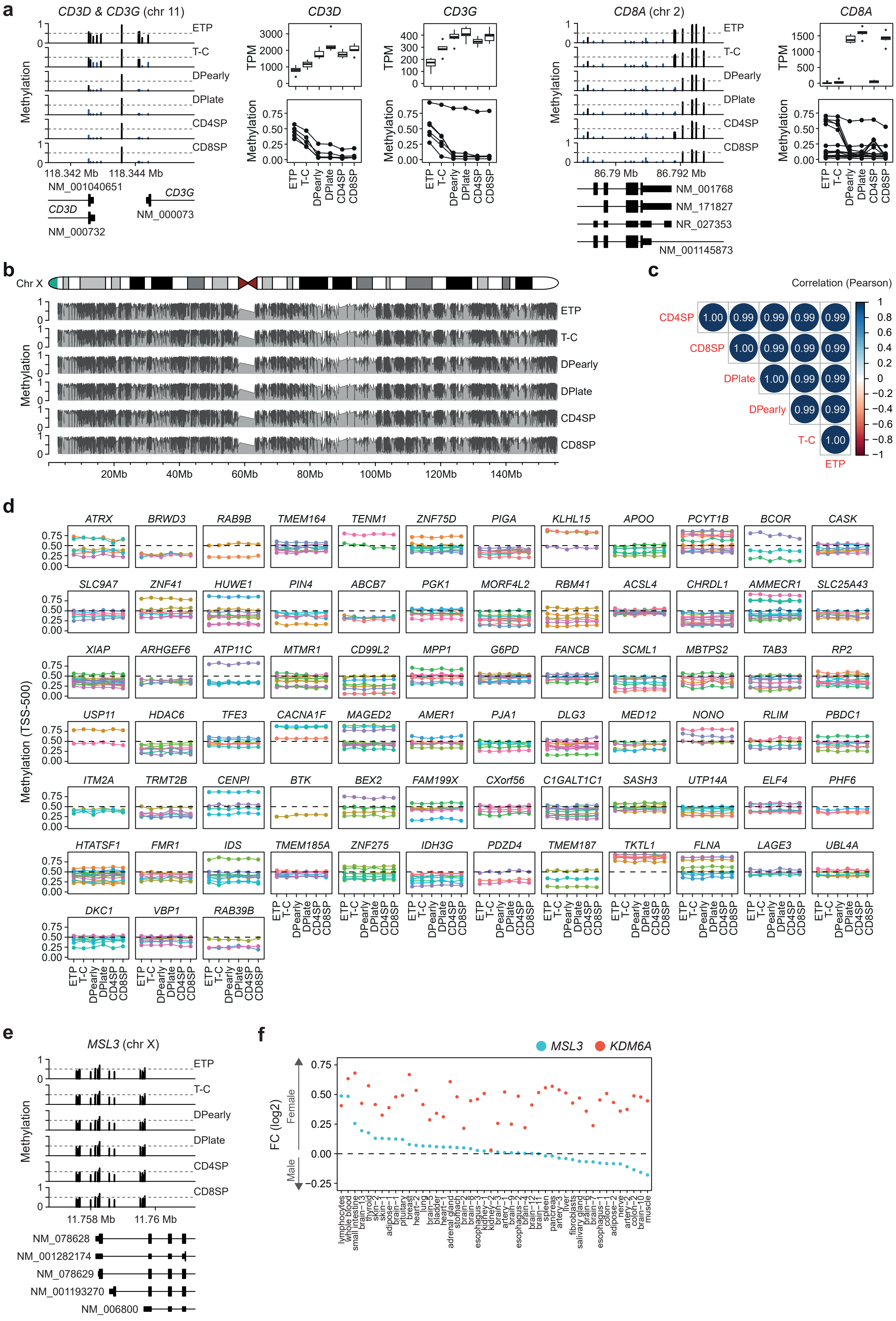
a
CD3D & CD3G (chr 11)
ETP
T-C
DPearly
DPlate
CD4SP
CD8SP
Methylation
118.342 Mb
118.344 Mb
NM_001040651
CD3D
NM_000732
CD3G
NM_000073
CD3D
CD3G
TPM
Methylation
CD8A (chr 2)
86.79 Mb
86.792 Mb
NM_001768
NM_171827
NR_027353
NM_001145873
CD8A
b
Chr X
20Mb
40Mb
60Mb
80Mb
100Mb
120Mb
140Mb
c
Correlation (Pearson)
d
Methylation (TSS-500)
ATRX
BRWD3
RAB9B
TMEM164
TENM1
ZNF75D
PIGA
KLHL15
APOO
PCYT1B
BCOR
CASK
SLC9A7
ZNF41
HUWE1
PIN4
ABCB7
PGK1
MORF4L2
RBM41
ACSL4
CHRDL1
AMMECR1
SLC25A43
XIAP
ARHGEF6
ATP11C
MTMR1
CD99L2
MPP1
G6PD
FANCB
SCML1
MBTPS2
TAB3
RP2
USP11
HDAC6
TFE3
CACNA1F
MAGED2
AMER1
PJA1
DLG3
MED12
NONO
RLIM
PBDC1
ITM2A
TRMT2B
CENPI
BTK
BEX2
FAM199X
CXorf56
C1GALT1C1
SASH3
UTP14A
ELF4
PHF6
HTATSF1
FMR1
IDS
TMEM185A
ZNF275
IDH3G
PDZD4
TMEM187
TKTL1
FLNA
LAGE3
UBL4A
DKC1
VBP1
RAB39B
e
MSL3 (chr X)
11.758 Mb
11.76 Mb
NM_078628
NM_001282174
NM_078629
NM_001193270
NM_006800
f
MSL3
KDM6A
FC (log2)
Female
Male
lymphocytes
whole blood
small intestine
brain-13
thyroid
skin-2
skin-1
adipose-1
brain-1
pituitary
breast
heart-2
lung
brain-5
bladder
heart-1
adrenal gland
stomach
brain-8
esophagus-3
kidney-2
brain-3
artery-1
brain-9
esophagus-2
brain-4
brain-12
brain-11
spleen
pancreas
artery-3
liver
fibroblasts
salivary gland
brain-6
brain-7
esophagus-1
colon-1
adipose-2
nerve
artery-2
colon-2
brain-10
muscle

Supplementary Figure 7: DNA methylation dynamics during human thymocyte development. a, DNA methylation around CD3D/CD3G and CD8A transcription start sites (TSS) (left), gene expression as transcript per million (TPM) (top) and DNA methylation of probes -500 and +1500 (bottom) in each thymocyte subpopulation. **b**, Stable X-chromosome DNA methylation across thymocyte subpopulations. **c**, Pearson correlation scores of X-chromosome methylation between thymocyte subpopulations. **d**, DNA methylation at TSS -500 of genes found to be inactive in both thymocytes and previous assessments across thymocyte development. Distinct colours for each probe from EPIC methylation array. **e**, DNA methylation of MSL3 at the TSS. **f**, Sex-biased expression as log2 fold change (FC) of expression in females over males of MSL3 and KDM6A across 46 GTEx tissues (n = 20; 10 male and 10 female for each tissue). **a,b,c,d**, n = 3 biological replicates (all female). Source data are provided as a Source Data file.

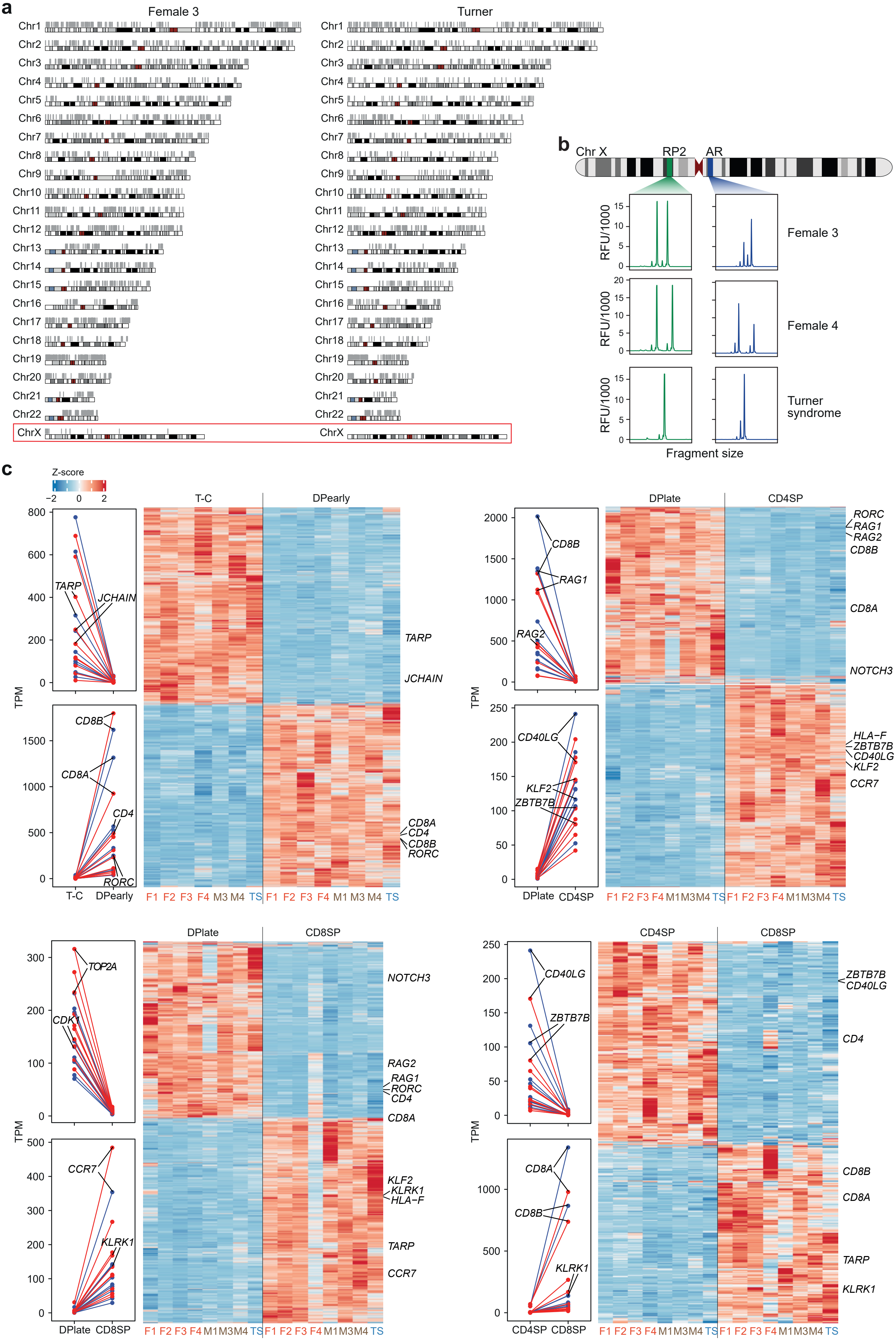
a
Female 3
Turner
Chr1
Chr2
Chr3
Chr4
Chr5
Chr6
Chr7
Chr8
Chr9
Chr10
Chr11
Chr12
Chr13
Chr14
Chr15
Chr16
Chr17
Chr18
Chr19
Chr20
Chr21
Chr22
ChrX
b
Chr X
RP2
AR
RFU/1000
Female 3
Female 4
Turner syndrome
Fragment size
c
Z-score
−2 0 2
TPM
T-C
DPearly
TARP
JCHAIN
CD8B
CD8A
CD4
RORC
F1 F2 F3 F4 M3 M4 TS
DPlate
CD4SP
RAG1
RAG2
NOTCH3
CD40LG
KLF2
ZBTB7B
HLA−F
CCR7
F1 F2 F3 F4 M1 M3 M4 TS
CD8SP
TOP2A
CDK1
KLRK1
KLF2

Supplementary Figure 8: Primary human thymocytes in Turner syndrome. a, Heterozygous SNPs identified in Female 3 (F3) and Turner Syndrome (TS) sample RNA-seq of CD4 single positive (CD4SP) cells. **b**, Repeat length of two polymorphic repeat loci in F3, F4 and the TS sample. **c**, Gene expression of transition genes, including the significant (Wald Test P-value < 1x10-3) genes with the highest fold change (log2) values between T-cell committed thymocytes (T-C) vs early double positive thymocytes (DPearly) (top left), late double positive thymocytes (DPlate) vs CD4SP (top right), DPlate vs CD8 single positive thymocytes (CD8SP) (bottom left) and CD4SP vs CD8SP (bottom right). Change in expression shown as transcript per million (TPM) for XX (n = 4) and XY (n = 3) karyotype (mean of all 7 samples; red) and TS (blue, n = 1) sample (line plot) and Z-score heatmap. Source data are provided as a Source Data file.

Short ID	Patient	Bulk RNA-seq	WES	EPIC array	Smart-seq2	qPCR	Note
M1	Male 1	X				X	
M2	Male 2					X	
M3	Male 3	X				X	
M4	Male 4	X					
F1	Female 1	X	X	X		X	
F2	Female 2	X	X			X	
F3	Female 3	X	X	X		X	cXCI
F4	Female 4	X	X	X	X		
TS	Turner Syndrome	X					

Supplementary Table 1: Sample overview.

Short ID	Patient	Cell type	Number sorted cells	Number reads RNA-seq (million)	Number reads WES (million)	Number cells passing filter Smart-seq2	Mean Number reads Smart-seq2
M3	Male 3	ETP	1500000	49			
M4	Male 4	ETP	1000000	54			
F1	Female 1	ETP	200000	44			
F2	Female 2	ETP	560000	39			
F3	Female 3	ETP	385000	66			
F4	Female 4	ETP	1000000	62		85	391844.941
TS	Turner Syndrome	ETP	270000	54			
M3	Male 3	T-C	850000	53			
M4	Male 4	T-C	300000	54			
F1	Female 1	T-C	300000	59			
F2	Female 2	T-C	1000000	35			
F3	Female 3	T-C	338000	43			
F4	Female 4	T-C	650000	61		84	365782.226
TS	Turner Syndrome	T-C	600000	62			
M1	Male 1	DPearly	1000000	49			
M3	Male 3	DPearly	1000000	42			
M4	Male 4	DPearly	1000000	50			
F1	Female 1	DPearly	2000000	48			
F2	Female 2	DPearly	1000000	42			
F3	Female 3	DPearly	1000000	46			
F4	Female 4	DPearly	1000000	64		84	377234.25
TS	Turner Syndrome	DPearly	1300000	53			
M1	Male 1	DPlate	1000000	45			
M3	Male 3	DPlate	1000000	39			
M4	Male 4	DPlate	1000000	53			
F1	Female 1	DPlate	2000000	54			
F2	Female 2	DPlate	1000000	45			
F3	Female 3	DPlate	1000000	44			
F4	Female 4	DPlate	1000000	64	127	72	490057.181
TS	Turner Syndrome	DPlate	1200000	54			
M1	Male 1	CD4SP	1000000	50			
M3	Male 3	CD4SP	1000000	46			
M4	Male 4	CD4SP	1000000	53			
F1	Female 1	CD4SP	2000000	30			
F2	Female 2	CD4SP	1000000	38			
F3	Female 3	CD4SP	1000000	48			
F4	Female 4	CD4SP	1000000	65		79	490611.684
TS	Turner Syndrome	CD4SP	1300000	55			
M1	Male 1	CD8SP	1000000	48			
M3	Male 3	CD8SP	1000000	41			
M4	Male 4	CD8SP	1000000	54			
F1	Female 1	CD8SP	660000	48			
F2	Female 2	CD8SP	1000000	44			
F3	Female 3	CD8SP	1000000	35			
F4	Female 4	CD8SP	1000000	68		69	289248.101
TS	Turner Syndrome	CD8SP	1000000	54			
F1	Female 1	PBMC			133		
F2	Female 2	PBMC			136		
F3	Female 3	PBMC			124		

Supplementary Table 2: Flow cytometry and sequencing metrics.

Name	Tissue details	Male samples (Suppl. Fig. 3b,e)	Female samples (Suppl. Fig. 3b,e)	Male samples (Suppl. Fig. 3c,d)	Female samples (Suppl. Fig. 3c,d)
adipose-1	Adipose_Subcutaneous	20	13	10	10
adipose-2	Adipose_Visceral_Omentum			10	10
artery-1	Artery_Aorta			10	10
artery-2	Artery_Coronary			10	10
artery-3	Artery_Tibial	43	32	10	10
brain-1	Brain_Amygdala			10	10
brain-2	Brain_Anterior_cingulate_cortex_BA24			10	10
brain-3	Brain_Caudate_basal_ganglia			10	10
brain-4	Brain_Cerebellar_Hemisphere			10	10
brain-5	Brain_Cerebellum			10	10
brain-6	Brain_Cortex	6	9	10	10
brain-7	Brain_Frontal_Cortex_BA9			10	10
brain-8	Brain_Hippocampus			10	10
brain-9	Brain_Hypothalamus			10	10
brain-10	Brain_Nucleus_accumbens_basal_ganglia			10	10
brain-11	Brain_Putamen_basal_ganglia			10	10
brain-12	Brain_Spinal_cord_cervical_c1			10	10
brain-13	Brain_Substantia_nigra			10	10
breast	Breast_Mammary_Tissue			10	10
salivary gland	Minor_Salivary_Gland			10	10
colon-1	Colon_Sigmoid			10	10
colon-2	Colon_Transverse			10	10
esophagus-1	Esophagus_Gastroesophageal_Junction			10	10
esophagus-2	Esophagus_Mucosa			10	10
esophagus-3	Esophagus_Muscularis			10	10
fibroblasts	Cells_Cultured_fibroblasts			10	10
heart-1	Heart_Atrial_Appendage			10	10
heart-2	Heart_Left_Ventricle	40	22	10	10
kidney-1	Kidney_Cortex			10	10
kidney-2	Kidney_Medulla			3	1
lymphocytes	Cells_EBV_transformed_lymphocytes			10	10
muscle	Muscle_Skeletal	53	33	10	10
nerve	Nerve_Tibial	32	22	10	10
skin-1	Skin_Not_Sun_Exposed_Suprapubic			10	10
skin-2	Skin_Sun_Exposed_Lower_leg	43	21	10	10
small intestine	Small_Intestine_Terminal_Ileum			10	10
adrenal gland	Adrenal_Gland			10	10
bladder	Bladder			7	7
liver	Liver			10	10
lung	Lung	45	30	10	10
pancreas	Pancreas			10	10
pituitary	Pituitary			10	10
spleen	Spleen			10	10
stomach	Stomach			10	10
thymocytes	Thymocytes			3	4
thyroid	Thyroid			10	10
whole blood	Whole_Blood	57	34	10	10

Supplementary Table 3: Tissue types analysed from public data.

Antibody	Clone	Cat. number	Dilution
Antibodies used for sorting PBMCs and T-cell populations from buffy coats			
CD3-FITC	UCHT1	300418 BioLegend	5 µl/10^6 cells in 100µl
CD8-APC	RPA-T8	555369 Beckton Dickinson	20 µl/10^6 cells in 100µl
Antibodies used for sorting of primary thymocytes with SH800			
CD4-FITC	RPA-T4	555346 Beckton Dickinson	4 µl/10^6 cells in 100 µl
CD8-APC-H7	SK1	560179 Beckton Dickinson	1.5 µl/10^6 cells in 100 µl
CD3-PE	UCHT1	555333 Beckton Dickinson	2.5 µl/10^6 cells in 100 µl
CD34-Biotin	SC136	130-113-177 Miltenyi Biotec	4 µl/10^6 cells in 100 µl
CD1a-PE	HI149	555807 Beckton Dickinson	5 µl/10^6 cells in 100 µl
Antibodies used for sorting of primary thymocytes with FACSAria Fusion			
CD4 - Per-Cy5.5	SK3	332772 Beckton Dickinson	5 µl/10^6 cells in 100 µl
CD8 - APC-H7	SK1	560179 Beckton Dickinson	2 µl/10^6 cells in 100 µl
CD3 – FITC	UCHT1	555332 Beckton Dickinson	5 µl/10^6 cells in 100µl
CD34 - biotin	SC136	130-113-177 Miltenyi Biotec	4 µl/10^6 cells in 100 µl
CD1a – PE	HI149	555807 Beckton Dickinson	5 µl/10^6 cells in 100 µl

Supplementary Table 4: Primary antibodies used for fluorescence-activated cell sorting.

Gene	Probe ID
CD99	Hs00908455_m1
CD40LG	Hs00163934_m1
DDX3X	Hs00606179_m1
EPAS1	Hs01026149_m1
KDM6A	Hs00253500_m1
P2RY8	Hs00736776_m1
USP9X	Hs00245009_m1
VAMP7	Hs00194568_m1
XIST	Hs01079824_m1
XIST	Hs03455388_s1
XIST	Hs01077162_m1

Supplementary Table 5: qPCR probes used.

Primer	Sequence (5’ – 3’)
AR_forward	ACCGAGGAGCTTTCCAGAAT
AR_reverse	GCTGCCTGGGGCTAGTCTCTT
RP2_forward	TGACATAGCGAGACCCTGTG
RP2_reverse	TGGTGGGTTCTCTAGCTGG

Supplementary Table 6: Oligonucleotides used in HUMARA assay and X-chromosome repeat amplification.
