# Supplementary material for: A landscape of X-inactivation during human T cell development
Source: Nat Commun. 2024 Dec 4;15:10527. doi: 10.1038/s41467-024-54110-7 (PMC11618795; doi:10.1038/s41467-024-54110-7)
Supplement: Supplementary file 2 — Description of Additional Supplementary Files [file 41467_2024_54110_MOESM2_ESM.pdf]

Description of Additional Supplementary Files

Supplementary Data 1. Thymocyte expression (transcript per million (TPM)) matrix. Related to Fig. 1b, 1c, 6b, 8a, 8b, 8c. Supplementary Fig. 2c, 7a and 8c.

Supplementary Data 2. Sleuth output from a likelihood ratio test (~Celltype vs ~1, two-tailed). Related to Supplementary Fig. 2c.

Supplementary Data 3. Male vs Female log2FC, Wald Test (two-tailed) p-values and BH-corrected q-values from Sleuth on combined thymocyte data. Positive values indicate female-bias, negative values indicate male-bias; related to Fig. 1e, 1f, 2c & Supplementary Fig. 3a, 3c, 3d.

Supplementary Data 4. Male vs Female log2FC, Wald Test (two-tailed) p-values and BH-corrected q-values from Sleuth on thymocyte subpopulations data. Positive values indicate female-bias, negative values indicate male-bias; related to Fig. 2a.

Supplementary Data 5. log2FC and empirical Bayes quasi-likelihood F-test (two-tailed) P-values obtained from edgeR (on STAR aligned and featureCounts processed data) for method comparison; related to Supplementary Fig. 3b (referred to as reference alignment).

Supplementary Data 6. Male vs Female log2fc, Wald Test (two-tailed) p-values and BH-corrected q-values from Sleuth for method comparison; related to Supplementary Fig. 3b (referred to as pseudo alignment).

Supplementary Data 7. Thymocyte allele-specific expression for positions on the X-chromosome in Female 3; related to Fig. 3c, 3d, 3e, 4b, 4c, Supplementary Fig. 4b, 4c & 5.

Supplementary Data 8. Female 4 Smart-seq2 allele-specific expression; related to Fig. 5a, 5b, 5c, Supplementary Fig. 6a, 6b, 6c and 6d.

Supplementary Data 9. Clustering results for each cell based on allelic expression of *XIST* and inactive genes (see methods under *Analysis of Smart-seq2* for details on how the clustering was performed); related to Fig. 5a, 5b, 5c, Supplementary Fig. 6a, 6b, 6c and 6d.

Supplementary Data 10. Gene, allele-specific expression (ASE) category (mean ASE across thymocyte subtypes < 0.4 = escape, ASE > 0.4 = inactive, no data = NA), Methylation_category (TSS -500 methylation < 0.25 = low, 0.25-0.75 = intermediate, > 0.75 = high) and sex-bias expression (log2FC, positive values indicate female-bias, negative values indicate male-bias); related to Fig. 7a.

Supplementary Data 11. gene, allele-specific expression categorization (see methods for detailed explanation of categories) in either thymocytes or previous assessments and comparison between classifications; related to Fig. 7b.
